# DRD4 Interacts with TGF‐β Receptors to Drive Colorectal Cancer Metastasis Independently of Dopamine Signaling Pathway

**DOI:** 10.1002/advs.202413953

**Published:** 2024-12-16

**Authors:** Yuan Zhou, Jinlong Tang, Menghan Weng, Honghe Zhang, Maode Lai

**Affiliations:** ^1^ Department of Pathology and Run Run Shaw Hospital Research Unit of Intelligence Classifification of Tumor Pathology and Precision Therapy, Chinese Academy of Medical Sciences (2019RU042) Zhejiang University School of Medicine Hangzhou Zhejiang 310058 China; ^2^ Department of Pathology the Second Affiliated Hospital of Zhejiang University School of Medicine Hangzhou Zhejiang 310058 China; ^3^ Department of Pathology the First Affiliated Hospital of Zhejiang University School of Medicine Hangzhou Zhejiang 310006 China; ^4^ Department of Pathology Research Unit of Intelligence Classification of Tumor Pathology and Precision Therapy Chinese Academy of Medical Sciences (2019RU042) Zhejiang University School of Medicine Hangzhou Zhejiang 310058 China

**Keywords:** DRD4, colorectal cancer, TGF‐β, TGFBR1, TGFBR2

## Abstract

The functional and pharmacological significance of dopamine receptor D4 (DRD4) in psychiatric and neurological disorders is well elucidated. However, the roles of DRD4 in colorectal cancer (CRC) remain unclear. This study observes a significant upregulation of DRD4 expression in clinical samples, which is negatively correlated with patient prognosis. In vitro, overexpression of DRD4 causes a constitutive activation of β‐Arrestin2/PP2A/AKT independent of dopamine. Interestingly, this classical signaling pathway is not associated with the phenotype of DRD4‐promoted migration and invasion in CRC cells. Instead, DRD4 interacts with transforming growth factor beta receptors (TGFBR1 and TGFBR2) to activate Smad2 phosphorylation and promote Smad2/Smad4 complex nucleus translocation. Then, *SNAI1* and *JAG1* are transcriptionally activated to induce epithelial‐mesenchymal transition and enhance the metastatic potential of CRC. Notably, the COOH‐terminal domain is identified as the key intracellular region for the pro‐metastatic roles of DRD4. Furthermore, treatment with a TGFBR1 inhibitor combined with a BMP inhibitor effectively counteracts the pro‐metastatic effects induced by DRD4 both in vitro and in vivo. In conclusion, these findings uncover an unconventional role for DRD4 beyond its classic function as a neurotransmitter receptor. The intracellular signaling of DRD4 interacting with TGFBR1 can be targeted pharmacologically for CRC therapy.

## Graphical abstract

This study identifies an unconventional role of dopamine receptor D4 (DRD4) in colorectal cancer (CRC), revealing its overexpression correlates with poor prognosis. DRD4 promotes metastasis by interacting with transforming growth factor beta receptors (TGFBR1 and TGFBR2) to activate Smad2 signaling and induce epithelial‐mesenchymal transition, independent of dopamine signaling. The COOH‐terminal domain is critical for this function. Blocking the DRD4‐TGFBR1 interaction effectively suppresses metastasis, suggesting a potential therapeutic strategy for CRC.

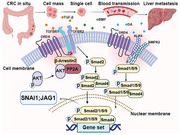

## 1 Introduction

Dopamine receptors including DRD1, DRD2, DRD3, DRD4, and DRD5 belong to the G protein‐coupled receptor (GPCR) family with a seven‐transmembrane structure. Based on their regulatory effects on adenylyl cyclase, they are further divided into two subfamilies.^[^ ^1^ ^]^ DRD1 and DRD5, as D1‐like receptors (D1R), activate the Gα_s/olf_ family of G proteins upon ligand binding, stimulating an increase in intracellular cyclic AMP (cAMP) levels. DRD2, DRD3, and DRD4, as D2‐like receptors (D2R), couple with the Gα_i/o_ family of G proteins, leading to a decrease in cAMP levels.^[^ ^2^, ^3^ ^]^ Neurotransmitter dopamine (DA) interacts with distinct dopamine receptors on cell membranes upon release from presynaptic terminals, mediating diverse physiological functions including reward, motivation, addiction, etc.^[^ ^4^, ^5^, ^6^, ^7^, ^8^, ^9^ ^]^

Among those dopamine receptors, DRD4 presents the lowest expression in the brain; however, DRD4 expression is observed in the retina, kidneys, adrenal glands, sympathetic ganglia, gastrointestinal tract, blood vessels, and heart.^[^ ^10^, ^11^ ^]^ In these organs, the physiological functions of DRD4 primarily rely on its activation in two distinct stages. At the first stage, when in its inactive state, DRD4 binds to Gα_i/o_ protein. Upon ligand or agonist binding, it becomes active, releasing active Gα_i_ protein that inhibits cAMP production.^[^ ^12^ ^]^ This leads to decreased protein kinase A (PKA) activity and subsequent phosphorylation of cAMP‐response element binding protein (CREB), regulating downstream target gene transcription.^[^ ^13^, ^14^ ^]^ At the second stage, beta‐Arrestin2 (β‐Arrestin2) acts as a signaling intermediary, involved in DA‐induced protein kinase B (AKT) inactivation independent of cAMP.^[^ ^15^ ^]^ Traditionally, β‐Arrestins are associated with GPCR signal termination and receptor internalization.^[^ ^16^, ^17^ ^]^ However, evidence from heterologous cell systems suggests that β‐Arrestins can also act as positive mediators of GPCR signaling independent of G proteins, via molecules like protein kinases.^[^ ^18^, ^19^ ^]^ In this context, DA binding to the DRD4 receptor triggers its activation, and the function of β‐Arrestin2 within this system involves the formation of a novel signaling complex that includes β‐Arrestin2, dephosphorylated AKT, and protein phosphatase 2A (PP2A), responding to DRD4 receptor activation, subsequently activating downstream glycogen synthase kinase‐3‐mediated signaling pathways.^[^ ^20^ ^]^

So far, it has been reported that DRD4 is abnormally expressed in various tumors. Besides nervous system tumors such as gliomas, glioblastoma, and pituitary tumors,^[^ ^21^, ^22^, ^23^ ^]^ DRD4 is also upregulated in breast cancer,^[^ ^24^, ^25^, ^26^ ^]^ non‐small cell lung cancer,^[^ ^27^ ^]^ prostate cancer,^[^ ^28^, ^29^, ^30^ ^]^ and colorectal cancer (CRC).^[^ ^31^, ^32^ ^]^ Moreover, overexpression of DRD4 is associated with tumor progression and prognosis. For instance, both breast and CRC patients with elevated DRD4 expression exhibit poor prognosis.^[^ ^24^, ^31^, ^32^ ^]^ In nervous system tumors and breast cancer, DRD4 functions as an oncogene that promotes tumor cell proliferation and metastasis.^[^ ^21^, ^24^, ^33^ ^]^ However, the elevated expression of DRD4 exhibits tumor‐suppressive functions in certain cancers. For example, DA activation of DRD4 in pancreatic cancer leads to a reduction in cAMP, subsequently inhibiting the activation of the PKA/p38 signaling pathway, thereby suppressing tumor‐associated macrophage‐mediated pro‐tumorigenic inflammation.^[^ ^34^ ^]^ Additionally, in pediatric central nervous system tumors the histone methyltransferase the enhancer of zeste homolog 2 inhibits DRD4 expression through enhancing the methylation of DRD4 promoter.^[^ ^35^ ^]^ Nevertheless, the underlying molecular mechanisms of DRD4 in tumor progression remains unclear. In this study, we investigate the expression and function of DRD4 in CRC and elucidate the DRD4‐regulated signaling pathway in CRC metastasis.

## 2 Results

### 2.1 DRD4 Functions a Pro‐Metastatic Role in CRC

**Figure 1:**
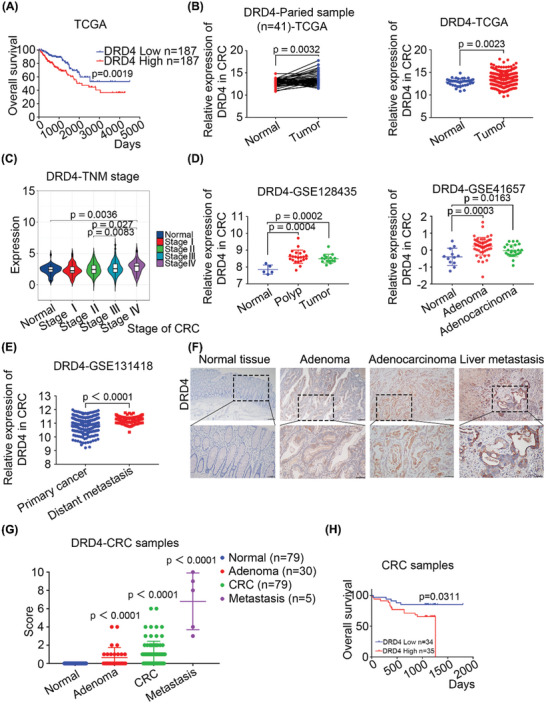
High expression of DRD4 leads to malignant progression of CRC. A) Kaplan–Meier plots of patients with CRC with high (*n* = 187) and low (*n* = 187) DRD4 expression. B) Expression of DRD4 in CRC paired and unpaired samples from TGCA database. Paired samples: (*n* = 41); Unpaired samples: Normal (*n* = 41); Tumor (*n* = 466). C) Expression of DRD4 in TNM stages of patients. Normal (*n* = 51); Stage I (*n* = 57); Stage II (*n* = 137); Stage III (*n* = 113); Stage IV (*n* = 52). D) GEO database: GSE128435 dataset and GSE41657 dataset represented the correlation between DRD4 and CRC progression. GSE128435: Normal (*n* = 5); Polyp (*n* = 22); Tumor (*n* = 15); GSE41657: Normal (*n* = 12); Adenoma (*n* = 51); Adenocarcinoma (*n* = 25). E). GEO database: GSE131418 dataset represented the correlation between DRD4 and CRC distant metastasis. GSE131418: Primary cancer (*n* = 332); Distant metastasis (*n* = 185). F) Representative images of standardized immunostaining for DRD4. G) Scores were assigned according to the results of (F): The positive area and intensity were evaluated double‐blind by two senior pathologists, and the evaluation results were assigned points according to the rules: positive index = positive area multiply by positive intensity. Assignment description: Positive area: <5%: zero points; 5–25%: one point; 26–50%: two points; 51–75%: three Points; >75%: four points; Positive intensity: negative (−), assigned zero points; Weak positive (+), assigned one point; Positive (++), assigned two points; Strong positive (+++), assigned three points; (+–++, 1.5 points; ++–+++, 2.5 points). Clinical samples: Normal (*n* = 79); Adenoma (*n* = 30); CRC (*n* = 79); Metastasis (*n* = 5). H) Kaplan–Meier plots of clinical CRC patients with high (*n* = 35) and low (*n* = 34) DRD4 expression. Data are presented as mean ± SD; statistical significance was assessed by an unpaired *t*‐test, paired *t*‐test, or one‐way ANOVA.

To screen the neurotransmitter receptors that regulate CRC progression, we analyzed the relationship between the expression of 16 GPCR receptors and the prognosis of CRC patients using. The Cancer Genome Atlas (TCGA) database (**Figure** **1**A; Figure S1A, Supporting Information). Among them, not only was high expression of DRD4 significantly correlated with poor prognosis (Figure 1A), but DRD4 was also markedly upregulated in CRC tissues compared to both paired and unpaired normal tissues (Figure 1B). The correlation of DRD4 with prognosis was further verified in the Gene Expression Omnibus (GEO) datasets GSE14333 and GSE38832 (Figure S1B, Supporting Information). Moreover, DRD4 expression increased progressively from tumor‐node‐metastasis (TNM) stage I to stage IV in CRC (Figure 1C). Interestingly, DRD4 expression was also significantly elevated in colorectal polyps, adenomas, and adenocarcinomas, as analyzed through the GEO databases GSE128435 and GSE41657 (Figure 1D). Additionally, GSE131418 dataset indicated that DRD4 was notably upregulated in CRC distant metastases compared to primary tumors (Figure 1E). To validate these online data, we assessed DRD4 expression in normal colorectal tissues, colorectal adenomas, CRC primary tumors, and liver metastases by immunohistochemistry (IHC) assay. As depicted in Figure 1F, DRD4 staining progressively intensified from normal tissues to adenomas, adenocarcinomas, and liver metastases. The statistical analysis results revealed that DRD4 expression was significantly higher in CRC tissues compared to 79 paired normal tissues and 30 paired adenoma tissues individually, and further increased in distant metastases (Figure 1G). In addition, we found that high expression of DRD4 was associated with poor prognosis in 69 cases with clinical prognostic information (Figure 1H). Multivariate Cox regression analysis indicated that DRD4 could be considered as an independent prognostic factor for survival in CRC patients (Table S1, Supporting Information). These clinical data demonstrate that DRD4 may be associated with CRC progression.

**Figure 2:**
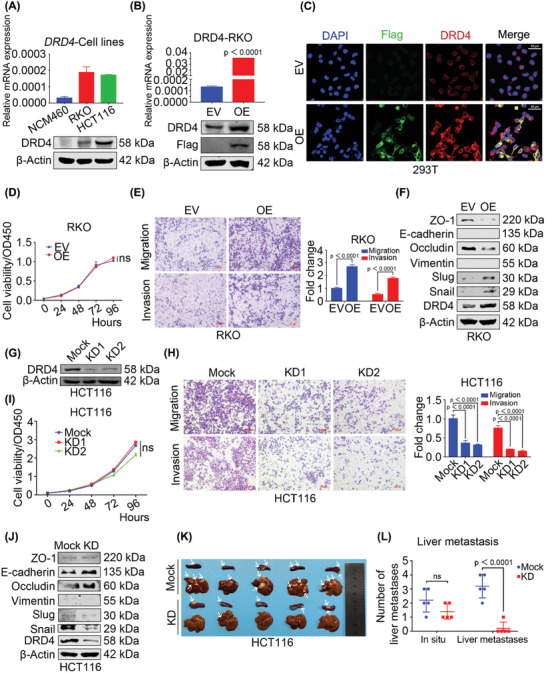
DRD4 promotes EMT process in vitro and metastasis in vivo. A) The mRNA levels and protein expression of DRD4 in NCM460, RKO, and HCT116 were examined by RT‐qPCR and WB. β‐Actin was run as an internal control. B) The mRNA levels and protein expression of endogenic and exogenous Flag‐tagged DRD4 were measured after stably transferred into pCDH‐DRD4‐N‐3×Flag plasmid by RT‐qPCR and WB. β‐Actin was run as an internal control in RKO cells. C) Immunofluorescence staining for endogenic (Alexa Fluor 546, red) and exogenous DRD4 with Flag‐tag (Alexa Fluor 488, green). Nuclei were stained with DAPI. Scale bars, 40 µm. D) Effect of overexpression of DRD4 on cell viability of RKO cells in 96 h. CCK8 test wavelength was 450 nm. E) Transwell assay to investigate the migratory and invasive properties of DRD4 in overexpressed RKO cells. The histograms on the right show the quantification analysis results. F) Protein levels of EMT markers were examined by WB in overexpressed RKO cells. β‐Actin was run as an internal control. G) WB to detect the expression of DRD4 in DRD4‐KD HCT116 cells. β‐Actin was run as an internal control in HCT116 cells. H) Transwell assay to investigate the migratory and invasive properties of DRD4‐KD HCT116 cells. The histograms on the right show the quantification analysis results. I) CCK8 assay was performed to detect cell viability of the DRD4‐KD HCT116 cells within 96 h. CCK8 test wavelength was 450 nm. J) Protein levels of EMT markers were examined by WB in DRD4‐KD HCT116 cells. β‐Actin was run as an internal control. K) Splenohepatic metastasis of DRD4‐Mock and DRD4‐KD HCT116 cells in nude mice. Mock (*n* = 5); KD (*n* = 5). L) Statistical diagram of the number of metastatic tumors in the livers of nude mice. Mock (*n* = 5); KD (*n* = 5). Data are presented as the mean ± SD; statistical significance was assessed by one‐way ANOVA, an unpaired *t*‐test, or two‐way ANOVA.

Next, we investigated the expression of dopamine receptors including DRD1, DRD2, DRD3, DRD4, and DRD5 in eight CRC cell lines, which showed that DRD4 was upregulated in these CRC cells compared to other dopamine receptor family members (Figure S2A, Supporting Information). Subsequently, we detected DRD4 expression in the normal colonic epithelial cell line (NCM460) and eight CRC cell lines using real‐time quantitative polymerase chain reaction (RT‐qPCR) and Western Blot (WB), which indicated that almost all CRC cell lines exhibited higher DRD4 levels than normal cell line. Specifically, among these CRC cell lines, SW620, HT29, and HCT116 carried a relatively high DRD4 expression, whereas SW480, HCT8, and RKO with a relatively low DRD4 expression (**Figure** **2**A; Figure S2B, Supporting Information). Moreover, immunofluorescence assay (IF) showed DRD4 primarily localized on the cell membrane of RKO, HCT116, and SW620 cells (Figure S2C, Supporting Information). To investigate the biological roles of DRD4 in CRC, we overexpressed DRD4 (DRD4‐OE) in RKO (Figure 2B) and HCT8 cells (Figure S2D, Supporting Information), confirming its correct localization to the cell membrane (Figure 2C). Overexpression of DRD4 did not affect cell proliferation (Figure 2D) but significantly promoted CRC cell migration and invasion (Figure 2E; Figure S2E, Supporting Information). Additionally, mesenchymal markers such as Slug and Snail were significantly increased, while the epithelial markers ZO‐1 and Occludin were decreased by DRD4 overexpression (Figure 2F). We further knocked down DRD4 (DRD4‐KD) in HCT116 and SW620 cells by Crispr/Cas9. Although complete deletion of DRD4 was not achieved due to multiple copies of the DRD4 gene in the genome, the expression of DRD4 was significantly reduced in both HCT116 (Figure 2G) and SW620 cells (Figure S2F, Supporting Information). Reduced DRD4 expression corresponded with decreased migratory and invasive capabilities of CRC cells (Figure 2H; Figure S2G, Supporting Information), with no significant change observed in cell proliferation (Figure 2I; Figure S2H, Supporting Information). Meanwhile, E‐cadherin and Occludin were increased and Snail was decreased in DRD4‐KD HCT116 cells (Figure 2J), while ZO‐1, E‐cadherin, and Occludin were increased and Vimentin was decreased in DRD4‐KD SW620 cells (Figure S2I, Supporting Information).

To further validate the pro‐metastatic role of DRD4 in vivo, we inoculated DRD4‐KD HCT116 cells into the spleen and subsequently monitored for liver metastasis formation 2 months later. The results showed that DRD4‐KD HCT116 cells formed fewer metastatic foci in the liver (Figure 2K,L; Figure S2J, Supporting Information).

### 2.2 DRD4 Promotes CRC Metastasis in a Dopamine‐Independent Manner

**Figure 3:**
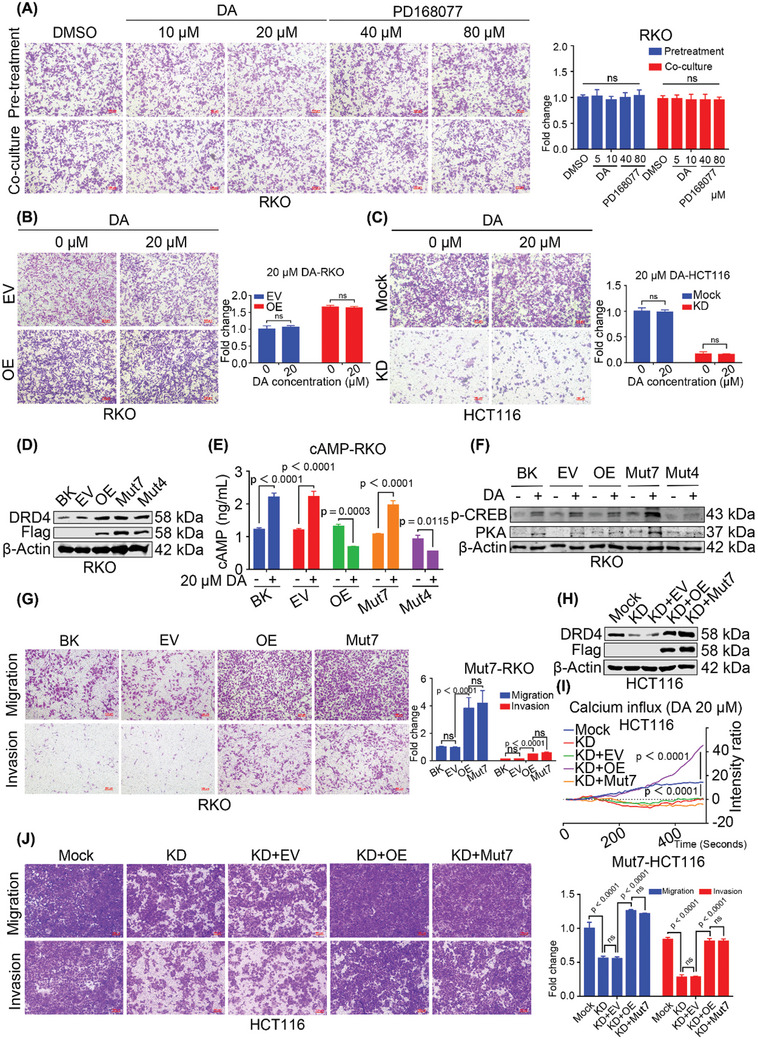
The function of promoting cancer metastasis of DRD4 is independent of DA. A) Transwell assay to investigate the migratory properties of DA and DRD4 agonist PD168077 in RKO cells with the treatment of pre‐culture (24 h) or co‐culture. The histograms on the right show the quantification analysis results. B) Transwell assay to investigate the migratory properties of 20 µm DA in DRD4‐overexpressing RKO cells. The histograms on the right show the quantification analysis results. C) Transwell assay to investigate the migratory properties of 20 µm DA in DRD4‐Mock and DRD4‐KD HCT116 cells. The histograms on the right show the quantification analysis results. D) Protein levels of endogenic and exogenous Flag‐tagged mutant DRD4 were examined by WB in exogenous DRD4 mutant RKO cells. β‐Actin was run as an internal control. E) ELISA assay to detect the effect of 20 µm DA on the cAMP concentrations of exogenous DRD4 mutant RKO cells in 6 h. F) Protein levels of p‐CREB and PKA were examined by WB in exogenous DRD4 mutant RKO cells. β‐Actin was run as an internal control. G) Transwell assay to investigate the migratory and invasive properties of full‐length and DA‐binding sites mutant DRD4 in wild‐type and DRD4‐Mut7 RKO cells. The histograms on the right show the quantification analysis results. H) WB to test endogenic and exogenous Flag‐tagged mutant DRD4 in the DRD4‐KD HCT116 cells. β‐Actin was run as an internal control. I) Fluorescence intensity change of the effect of 20 µm DA on the full‐length and DA‐binding sites mutant DRD4 in the DRD4‐KD HCT116 cells with calcium probe incubation. J) Transwell assay to investigate the migratory and invasive properties of full‐length and DA‐binding sites mutant DRD4 in the DRD4‐KD HCT116 cells. The histograms on the right show the quantification analysis results. Data are presented as the mean ± SD; statistical significance was assessed by two‐way ANOVA.

To explore whether DA is required for DRD4 to exert its pro‐metastatic role in CRC, we initially measured DA levels by ELISA, which revealed lower DA levels in both the supernatants and cell lysis of CRC cells compared to normal colonic epithelial cells (Figure S3A, Supporting Information). Interestingly, neither DA nor the DRD4 receptor agonist PD168077 increased RKO cell migration capability, whether applied as pre‐treatment or co‐culture (**Figure** **3**A). Although cell viability increased along with increasing DA concentrations, no differences were observed in either DRD4‐overexpressing RKO cells (Figure S3B, Supporting Information) or DRD4‐KD HCT116 cells (Figure S3C, Supporting Information). Cell proliferation assay also indicated no difference between DRD4‐overexpressing and control RKO cells with DA treatment (Figure S3D, Supporting Information), and a finding similarly observed in DRD4‐KD HCT116 cells (Figure S3E, Supporting Information). Notably, even under DA treatment, DRD4 overexpression could still promote CRC cell migration (Figure 3B), whereas DRD4 knockdown inhibited CRC cell migration (Figure 3C). These results demonstrate that the promotion of CRC metastasis by DRD4 may be independent of DA presence or absence.

A previous study has predicted the binding sites of DRD4 to DA,^[^ ^36^ ^]^ so we constructed two DRD4 mutants: one with seven mutations (D115A, L187A, Y192A, V193A, S196A, S197A, and S200A. Named as Mut7) and another with four mutations (D115A, V116A, C119A, and T120A. Named as Mut4) (Figure 3D; Figure S3F, Supporting Information). As we know, activation of D1Rs typically increases cAMP levels, while DRD4 activation, a D2R, suppresses cAMP.^[^ ^37^ ^]^ However, the DRD4 mutants with seven mutations (DRD4‐Mut7) caused a significant elevation of cAMP, but the DRD4 mutants with four mutations (DRD4‐Mut4) still decreased cAMP level (Figure 3E). As the downstream signals, PKA and p‐CREB expression were also increased by DRD4‐Mut7, yet not by DRD4‐Mut4 (Figure 3F). These findings indicate that the seven amino acid sites are essential for the binding of DRD4 to DA, while mutations at these sites disrupt their interaction. Furthermore, the transwell assay showed that the DRD4‐Mut7 still promoted CRC cell migration and invasion as same as wild‐type DRD4 (Figure 3G). The increased intracellular calcium influx could also serve as a marker of DRD4 activation.^[^ ^37^, ^38^ ^]^ When both wild‐type and Mut7 DRD4 were re‐expressed in DRD4‐KD HCT116 cells (Figure 3H), intracellular calcium influx was recovered by expression of wild‐type DRD4, but not by Mut7 DRD4 (Figure 3I). However, not only wild‐type but also Mut7 DRD4 could rescue the migration and invasion capabilities of DRD4‐KD HCT116 cells (Figure 3J). Taken together, these findings suggest that the promotion of CRC cell migration and invasion by DRD4 is independent of DA signaling activation.

### 2.3 DRD4 Functions as Pro‐Metastatic Roles Depending on the COOH‐Terminal Domain but not Through the Persistent Late‐Stage Activation State

**Figure 4:**
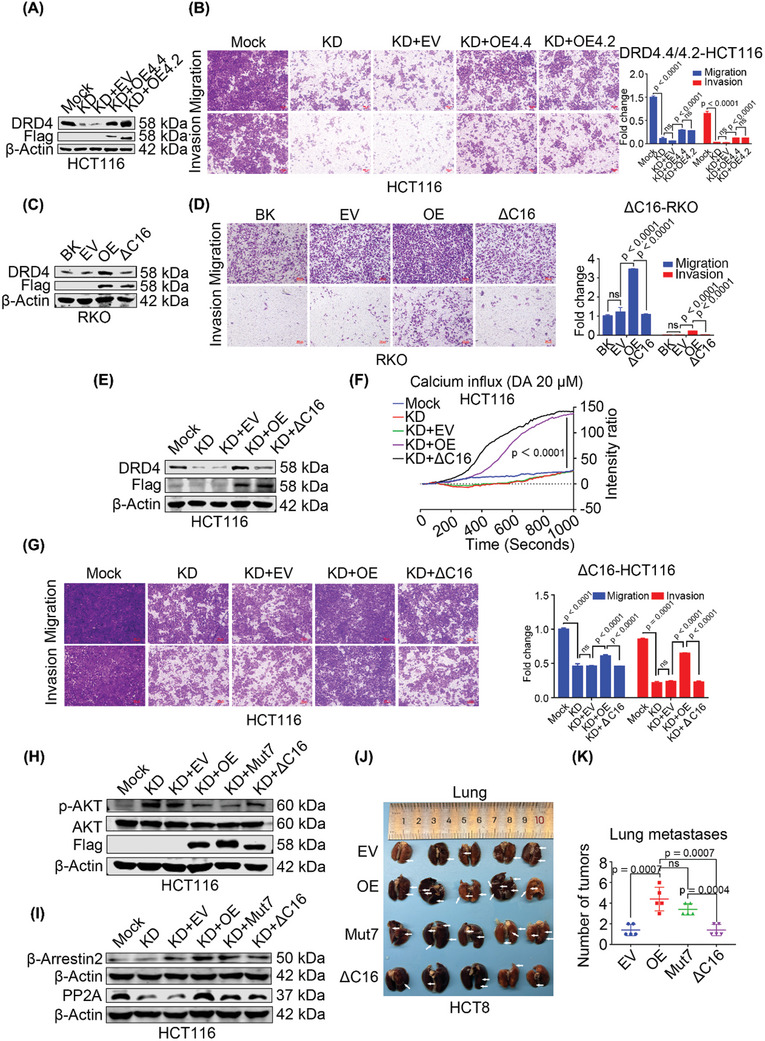
The non‐DA‐dependent constitutive activation of DRD4 is dependent on the COOH terminal. A) WB to detect exogenous Flag‐tagged mutant DRD4 with four (DRD4‐OE4.4) or two (DRD4‐OE4.2) VNTR regions in DRD4‐KD HCT116 cells. β‐Actin was run as an internal control. B) Transwell assay to investigate the migratory and invasive properties of exogenous Flag‐tagged mutant DRD4 with four (DRD4‐OE4.4) or two (DRD4‐OE4.2) VNTR regions in DRD4‐KD HCT116 cells. The histograms on the right show the quantification analysis results. C) WB to detect DRD4 with full‐length or DRD4‐ΔC16 in RKO cells. β‐Actin was run as an internal control. D) Transwell assay to investigate the migratory and invasive properties of DRD4 with full‐length or DRD4‐ΔC16 in RKO cells. The histograms on the right show the quantification analysis results. E) WB to detect exogenous Flag‐tagged mutant DRD4 with full‐length or DRD4‐ΔC16 in DRD4‐KD HCT116 cells. β‐Actin was run as an internal control. F) Fluorescence intensity change of the effect of 20 µm DA on the DRD4 with full‐length or DRD4‐ΔC16 in DRD4‐KD HCT116 cells with calcium probe incubation. G) Transwell assay to investigate the migratory and invasive properties of DRD4 with full‐length or DRD4‐ΔC16 in DRD4‐KD HCT116 cells. The histograms on the right show the quantification analysis results. H) WB to detect the levels of p‐AKT with exogenous Flag‐tagged mutant DRD4 in DRD4‐KD HCT116 cells. Total AKT was run as an internal control. I) WB to detect the levels of β‐Arrestin2 and PP2A with exogenous Flag‐tagged mutant DRD4 in DRD4‐KD HCT116 cells. β‐Actin was run as an internal control. J) Representative images of metastatic lungs. EV (*n* = 5); OE (*n* = 5); Mut7 (*n* = 5); ΔC16 (*n* = 5). The white arrows represent the tumors. K) Statistical diagram of the number of metastatic tumors in the lungs of NCG mice injected via tail vein. EV (*n* = 5); OE (*n* = 5); Mut7 (*n* = 5); ΔC16 (*n* = 5). Data are presented as the mean ± SD; statistical significance was assessed by two‐way ANOVA or an unpaired *t*‐test.

There are two crucial structural‐functional domains in DRD4: one located in the third intracellular loop with a 48‐bp variable number tandem repeat (VNTR), and the other at the palmitoylation site near the COOH‐terminal end. Notably, the third cytoplasmic loop of DRD4 encodes a VNTR tract comprising a 16‐amino acid segment, with 2–11 repeat variants.^[^ ^39^ ^]^ To investigate whether DRD4 promotes CRC metastasis through the variant VNTR, we produced DRD4‐overexpressing vectors with two or four VNTR repeats (**Figure** **4**A). These results showed that DRD4 with either two or four VNTR repeats could rescue migration and invasion in DRD4‐KD HCT116 cells (Figure 4B), implying that the VNTR region is not the crucial structural domain for DRD4 in promoting the metastasis of CRC. Conversely, overexpression of DRD4 with a 16‐amino acids deletion at the COOH‐terminal end (DRD4‐ΔC16) in RKO cells failed to enhance migration and invasion capabilities (Figure 4C,D). Subsequent overexpression of DRD4‐ΔC16 in DRD4‐KD HCT116 cells (Figure 4E) restored calcium influx levels similar to wild‐type DRD4 (Figure 4F), without affecting DA binding capacity. However, DRD4‐ΔC16 was not as capable of rescuing DRD4‐KD HCT116 cell migration and invasion as wild‐type DRD4 (Figure 4G).

As a D2‐type dopamine receptor, DRD4 activation by DA includes early and late states. p‐AKT dephosphorylation occurs in the late stage and depends on heterotrimer formation with PP2A and β‐Arrestin2.^[^ ^20^ ^]^ As expected, the level of phosphorylated AKT significantly increased in CRC cells following DA treatment (Figure S4A, Supporting Information). However, overexpression of DRD4 decreased phosphorylated AKT levels, while DRD4 knockdown increased phosphorylated AKT levels (Figure S4B, Supporting Information). Interestingly, regardless of DA treatment, DRD4 overexpression could consistently lead to a decrease of phosphorylated AKT in CRC cells (Figure S4C, Supporting Information). Furthermore, both wild‐type DRD4 and DRD4‐Mut7 decreased phosphorylated AKT levels, whereas DRD4‐ΔC16 did not (Figure 4H). Accordingly, β‐Arrestin2 and PP2A were up‐regulated by wild‐type DRD4 and DRD4‐Mut7 but not by DRD4‐ΔC16 (Figure 4I). Given that many previous studies have confirmed AKT signal activation could promote CRC epithelial‐mesenchymal transition (EMT) and metastasis, DRD4 functioned as a pro‐metastatic role not through activating late states DA signal. Further, the ability of DRD4‐ΔC16 to inhibit migration and invasion capabilities was reconfirmed in vitro (Figure S4D, Supporting Information), and in vivo tail vein injection model showed that DRD4‐ΔC16 reduced the lung (Figure 4J,K) but not kidney (Figure S4E,F, Supporting Information) metastasis capacity of HCT8 cells (Figure S4G, Supporting Information). Together, the COOH‐terminal domain of DRD4 simultaneously mediates CRC metastasis and late states DRD4 activation, while this latter activation is not linked to its migratory and invasive functions.

### 2.4 DRD4 Activates the TGF‐β Signaling Pathway

**Figure 5:**
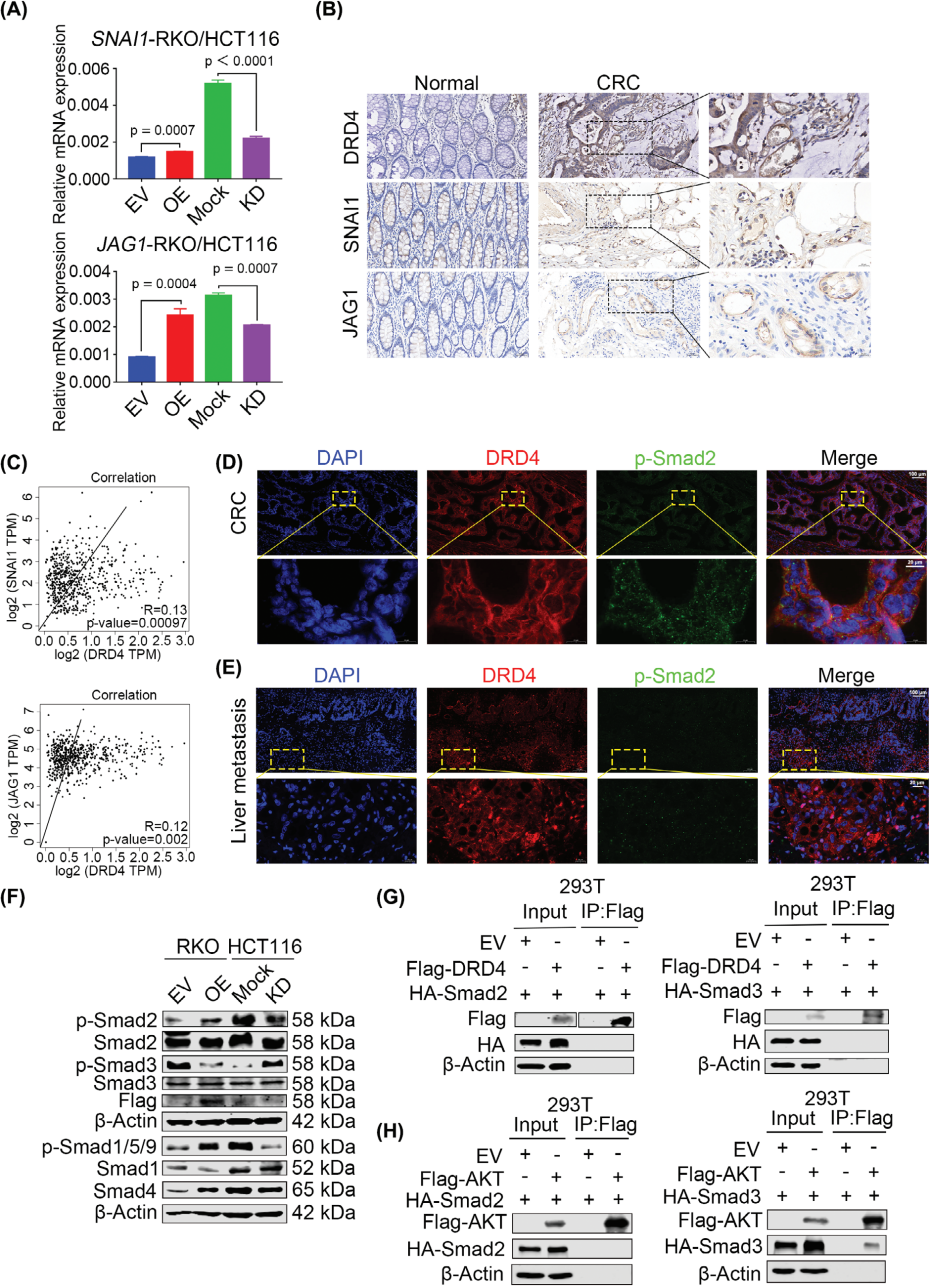
Constitutive activation of DRD4 is associated with the TGF‐β signaling pathway. A) The mRNA levels of *SNAI1* and *JAG1* in DRD4‐overexpressing RKO and DRD4‐KD HCT116 cells were examined by RT‐qPCR. β‐Actin was run as an internal control. B) Representative images of standardized immunostaining for DRD4, SNAI1, and JAG1 in clinical CRC samples. C) Correlation analysis of TCGA database SNAI1 and DRD4, as well as JAG1 and DRD4. D) Immunofluorescence staining for DRD4 (Alexa Fluor 546, red) and p‐Smad2 (Alexa Fluor 488, green). Nuclei were stained with DAPI. Scale bars, 100 µm; 20 µm. E) Immunofluorescence staining for DRD4 (Alexa Fluor 546, red) and p‐Smad2 (Alexa Fluor 488, green). Nuclei were stained with DAPI. Scale bars, 100 µm; 20 µm. F) WB to detect the levels of p‐Smad2, Smad2, p‐Smad3, Smad3, p‐Smad1/5/9, Smad1, and Smad4 in the DRD4‐overexpressing RKO and DRD4‐KD HCT116 cells. Smad2, Smad3, Smad1, or β‐Actin was run as an internal control. G) Immunoblotting to detect the immunoprecipitation of exogenous Flag‐tagged DRD4 and HA‐tagged Smad2 or Smad3 by an anti‐Flag antibody in 293T cells. To prevent overexposure, images are displayed in different exposures and the same below. H) Immunoblotting to detect the immunoprecipitation of exogenous Flag‐tagged AKT with HA‐tagged Smad2 or Smad3 by an anti‐Flag antibody in 293T cells, respectively. Data are presented as the mean ± SD; statistical significance was assessed by an unpaired *t*‐test.

To elucidate which pathway activated by DRD4 contributes to CRC metastasis, we conducted RNA‐seq in DRD4‐KD HCT116 cells. The results revealed enrichment of genes associated with TGF‐β‐related signaling pathways (Figure S5A, Supporting Information). RT‐qPCR confirmed that snail family transcriptional repressor 1 (*SNAI1*) and Jagged1 (*JAG1*), among others, downstream target genes of the TGF‐β pathway, were upregulated by DRD4 overexpression and downregulated by DRD4 knockdown (**Figure** **5**A; Figure S5B,C, Supporting Information). Additionally, IHC results displayed a significant positive correlation between DRD4 and SNAI1, as well as DRD4 and JAG1 expression in CRC samples (Figure 5B,C). Furthermore, IF assays revealed co‐localization of p‐Smad2 and SNAI1, as the activation signaling of TGF‐β pathway, which were highly expressed with DRD4 in the clinical CRC samples (Figure 5D; Figure S5D, Supporting Information), and p‐Smad2 was also expressed in the clinical liver metastasis samples with high DRD4 expression (Figure 5E). Otherwise, p‐Smad2, p‐Smad1/5/9, and Smad4 levels were increased with DRD4 overexpression and decreased with DRD4 knockdown (Figure 5F), which was verified in HCT8 cells (Figure S5E, Supporting Information). In the liver metastases of mice with high DRD4 expression, the elevated expression of p‐Smad2, p‐Smad1/5/9, and SNAI1 was also observed (Figure S5F,G, Supporting Information). Unexpectedly, p‐Smad3 levels decreased when DRD4 was overexpressed, conversely, increased upon DRD4 knockdown (Figure 5F). Exogenous co‐immunoprecipitation (Co‐IP) results showed no interaction of DRD4 and Smad2 or Smad3 (Figure 5G).

As we know, the phosphorylation of Smad3 has traditionally been considered essential in the classical TGF‐β and BMP pathways. However, we used AKT phosphorylation inhibitor, perifosine (Peri), to treat DRD4‐KD HCT116 cells and SW620, which showed that suppressing AKT phosphorylation increased p‐Smad2 and p‐Smad1/5/9 levels but decreased p‐Smad3 levels (Figure S6A,C,E,G, Supporting Information). Conversely, the AKT phosphorylation activator SC79 reduced p‐Smad2 and p‐Smad1/5/9 expression while increasing p‐Smad3 levels (Figure S6B,D,F,H, Supporting Information). Furthermore, AKT could interact with Smad3, but not Smad2 (Figure 5H). These data elucidate that DRD4 activates Smad2 and Smad1/5/9 phosphorylation, but inhibits Smad3 phosphorylation through repressing the AKT pathway.

### 2.5 DRD4 Interacts with TGF‐β Family Receptors

**Figure 6:**
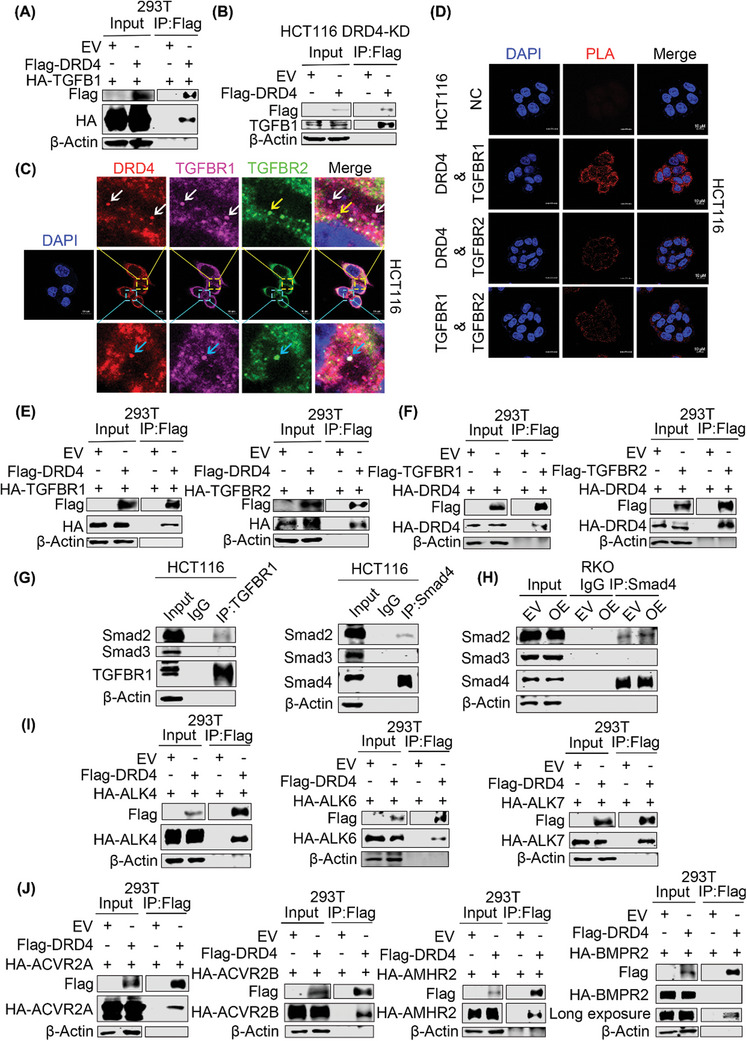
DRD4 interacts with TGF‐β family receptors. A) Immunoblotting to detect the immunoprecipitation of exogenous Flag‐tagged DRD4 and HA‐tagged TGFB1 by an anti‐Flag antibody in 293T cells. B) Immunoblotting to detect the immunoprecipitation of exogenous Flag‐tagged DRD4, endogenous TGFB1 by an anti‐Flag antibody in the DRD4‐KD HCT116 cells. C) Immunofluorescence staining for DRD4 (Alexa Fluor 546, red), TGFBR1 (Alexa Fluor 647, purple) and TGFBR2 (Alexa Fluor 488, green) in HCT116 cells. Nuclei were stained with DAPI. Scale bars, 10 µm. The white arrows represent the co‐location of DRD4 and TGFBR1. The yellow arrows represent the solitary presence of TGFBR2. The blue arrows represent the co‐positioning of DRD4, TGFBR1, and TGFBR2. D) PLA was performed to detect the interaction of DRD4, TGFBR1, and TGFBR2 in HCT116 cells. Alexa Fluor 546‐labeled positive sites (red). Nuclei were stained with DAPI. Scale bars, 10 µm. E) Immunoblotting to detect the immunoprecipitation of exogenous Flag‐tagged DRD4 with HA‐tagged TGFBR1 or HA‐tagged TGFBR2 by an anti‐Flag antibody in 293T cells, respectively. F) Immunoblotting to detect the immunoprecipitation of exogenous Flag‐tagged TGFBR1 or TGFBR2 with HA‐tagged DRD4 by an anti‐Flag antibody in 293T cells, respectively. G) Immunoblotting to detect the immunoprecipitation of endogenous TGFBR1 or Smad4 with Smad2 or Smad3 by an anti‐TGFBR1 or anti‐Smad4 antibody in HCT116 cells, respectively. H) Immunoblotting to detect the immunoprecipitation of endogenous Smad4 with Smad2 or Smad3 by an anti‐Smad4 antibody in DRD4‐overexpressing RKO cells, respectively. I) Immunoblotting to detect the immunoprecipitation of exogenous Flag‐tagged DRD4 with HA‐tagged ALK4, ALK6, or ALK7 by an anti‐Flag antibody in 293T cells, respectively. J) Immunoblotting to detect the immunoprecipitation of exogenous Flag‐tagged DRD4 and HA‐tagged ACVR2A, ACVR2B, AMHR2, or BMPR2 by an anti‐Flag antibody in 293T cells, respectively.

To investigate how DRD4 activates the TGF‐β pathway, we conducted Co‐IP experiments in 293T cells, which showed an interaction between DRD4 and TGFB1 (**Figure** **6**A). In DRD4 knockdown HCT116 cells, exogenous DRD4 could also pull down TGFB1 (Figure 6B). These results suggest that DRD4 may be considered a TGF‐β receptor or may form an indirect complex with it. IF assays showed co‐localization of DRD4 with transforming growth factor beta receptor 1 (TGFBR1) and transforming growth factor beta receptor 2 (TGFBR2) in HCT116 cells (Figure 6C) and clinical CRC samples (Figure S7A, Supporting Information). Proximity ligation assay (PLA) signal amplification experiments in HCT116 cells revealed a strong interaction between DRD4 and both TGFBR1 and TGFBR2, but a relatively weak interaction between TGFBR1 and TGFBR2 (Figure 6D). Moreover, the exogenous reciprocal interaction of DRD4 with TGFBR1 and TGFBR2 was confirmed in 293T cells (Figure 6E,F). However, DRD4‐ΔC16 did not reduce the interaction between DRD4 and TGFBR1 or TGFBR2 (Figure S7B, Supporting Information). Instead, it decreased the phosphorylation of TGFBR1 and TGFBR2 by reducing the transmission of phosphorylation (Figure S7C, Supporting Information), thereby inhibiting the activation of the TGF‐β signaling pathway. Intriguingly, TGFBR1 was observed to bind to Smad2 but not Smad3 in HCT116 cells, and Smad4 was also found to bind only to Smad2 (Figure 6G), suggesting a ligand‐independent interaction. The aforementioned results indicate that activated TGFBR1 phosphorylates downstream Smad2, facilitating the formation of the Smad2‐Smad4 complex. Consistent with this, DRD4 overexpression enhanced the interaction between smad2 and smad4 in the absence of ligands (Figure 6H). To explore whether DRD4 could bind to other members of the TGF‐β family, molecular docking predictions (Figure S8A, Supporting Information) and Co‐IP assay further verified the interaction of DRD4 with 4 type‐I receptors (4/7) of TGF‐β family including ALK4, ALK5 (TGFBR1), ALK6, and ALK7 (Figure 6E,I; Figure S8B, Supporting Information), as well as with all five type‐II receptors, including TGFBR2, ACVR2A, ACVR2B, AMHR2, and BMPR2 (Figure 6E,J). Meanwhile, molecular docking predictions between DRD4‐ΔC16 and TGF‐β family receptors reconfirmed that the loss of COOH terminal did not affect molecular binding but reduced TGF‐β signaling through phosphorylation (Figure S8C, Supporting Information). These results underscore the important role of DRD4 in regulating the TGF‐β pathway.

### 2.6 DRD4 Induces Ligand‐Independent Activation of TGF‐β Pathway to Promote CRC Metastasis

**Figure 7:**
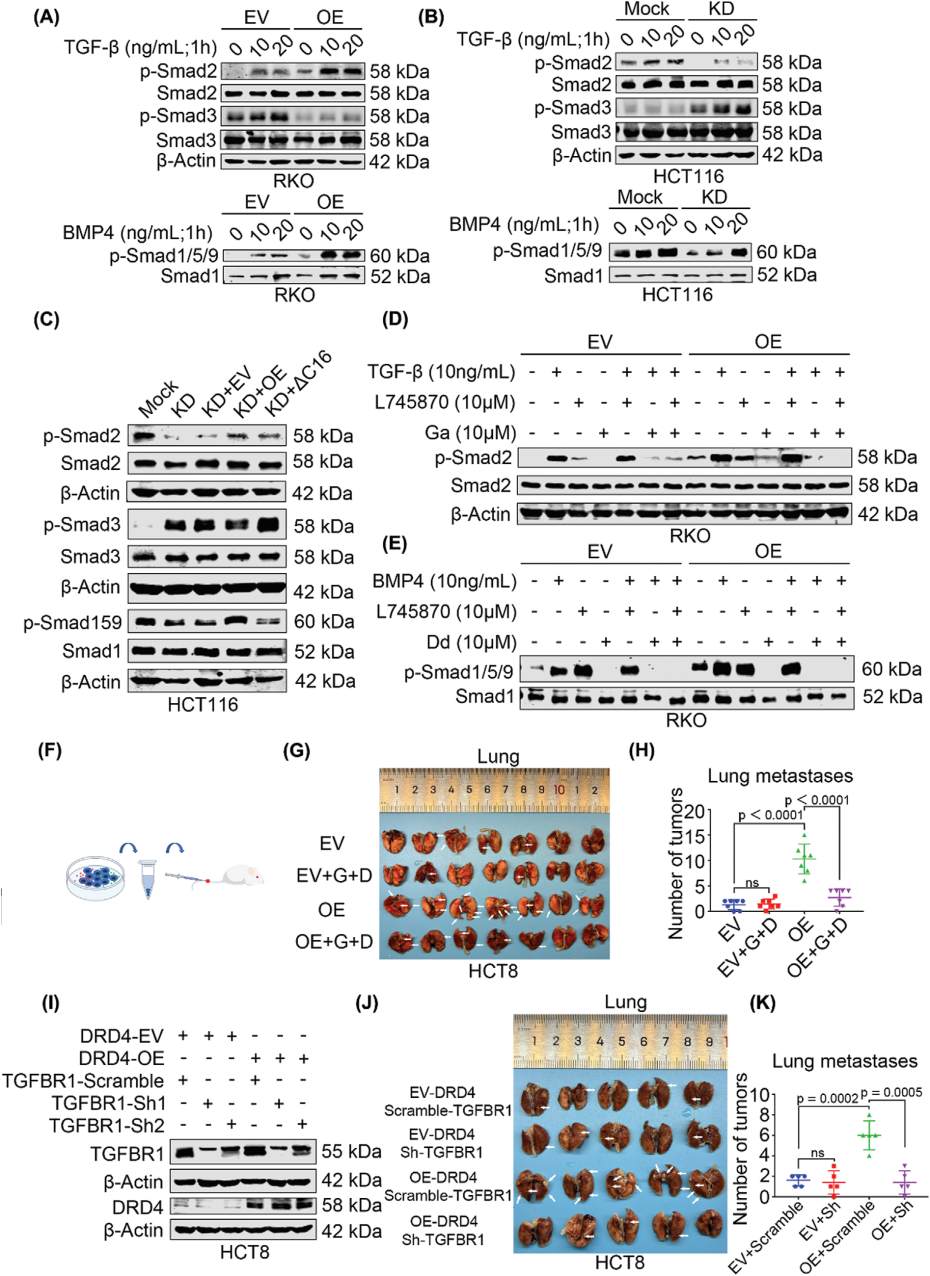
Inhibition of TGF‐β receptors inhibits DRD4‐induced metastasis. A) WB to detect the effect of concentration gradient of TGF‐β or BMP4 on p‐Smad2 and p‐Smad3 or p‐Smad1/5/9 in DRD4‐overexpressing RKO cells at 24 h, respectively. Smad2, Smad3, or Smad1 was run as an internal control. B) WB to detect the effect of concentration gradient of TGF‐β or BMP4 on p‐Smad2 and p‐Smad3 or p‐Smad1/5/9 in DRD4‐KD HCT116 cells at 24 h, respectively. Smad2, Smad3, or Smad1 was run as an internal control. C) WB to detect the effect of wile‐type DRD4 and DRD4‐ΔC16 on p‐Smad2, p‐Smad3, and p‐Smad1/5/9 in DRD4 knockdown HCT116 cells, respectively. Smad2, Smad3, or Smad1 was run as an internal control. D) WB to detect the effect of DRD4 inhibitor L745870 and TGFBR1 kinase inhibitor Ga on p‐Smad2 in DRD4‐overexpressing RKO cells. Smad2 was run as an internal control. E) WB to detect the effect of DRD4 inhibitor L745870 and BMP receptors (ALK2, ALK3, and ALK6) inhibitor Dd on p‐Smad1/5/9 in DRD4‐overexpressing RKO cells. Smad1 was run as an internal control. F) Animal model diagram. G) Representative images of metastatic lungs. EV (*n* = 7); EV+G+D (*n* = 7); OE (*n* = 7); OE+G+D (*n* = 7). The white arrows represent the tumors. H) Statistical diagram of the number of metastatic tumors in the lungs of NCG mice injected via tail vein. EV (*n* = 7); EV+G+D (*n* = 7); OE (*n* = 7); OE+G+D (*n* = 7). I) WB to detect the knockdown efficiency of TGFBR1 in DRD4‐overexpressing HCT8 cells. β‐Actin was run as an internal control. J) Representative images of metastatic lungs. EV‐DRD4+Scramble‐TGFBR1 (*n* = 5); EV‐DRD4+Sh‐TGFBR1 (*n* = 5); OE‐DRD4+Scramble‐TGFBR1 (*n* = 5); OE‐DRD4+Sh‐TGFBR1 (*n* = 5). The white arrows represent the tumors. K) Statistical diagram of the number of metastatic tumors in the lungs of NCG mice injected via tail vein. EV+Scramble (*n* = 5); EV+Sh (*n* = 5); OE+Scramble (*n* = 5); OE+Sh (*n* = 5). Data are presented as the mean ± SD; statistical significance was assessed by an unpaired *t*‐test.

First, we treated RKO cells with varying concentrations of recombinant TGF‐β and BMP4 at different time points, which showed that TGF‐β dramatically increased p‐Smad2 and p‐Smad3 levels (Figure S9A,B, Supporting Information), while BMP4 activated p‐Smad1/5/9 (Figure S9C,D, Supporting Information). In DRD4 overexpressing RKO cells treated with TGF‐β and BMP4, DRD4 further enhanced Smad2 and Smad1/5/9 phosphorylation levels and inhibited Smad3 phosphorylation (**Figure** **7**A). Conversely, DRD4 knockdown decreased the Smad2 and Smad1/5/9 phosphorylation levels activated by TGF‐β and BMP4 and increased the Smad3 phosphorylation (Figure 7B). Subsequently, wild‐type DRD4 and DRD4‐ΔC16 were re‐expressed in DRD4‐KD HCT116 cells, and the results showed that wild‐type DRD4 but not DRD4‐ΔC16 activated p‐Smad2 and p‐Smad1/5/9, inhibited p‐Smad3, and COOH‐terminal domain of DRD4 was key to activating the TGF‐β pathway (Figure 7C).

Interestingly, the DRD4 inhibitor L745870 could not reduce Smad2 and Smad1/5/9 phosphorylation levels. In contrast, the TGFBR1 kinase inhibitor Galunisertib (Ga) significantly inhibited Smad2 phosphorylation (Figure 7D; Figure S9E,F, Supporting Information). Similar results were observed with the BMP receptors (ALK2, ALK3, and ALK6) inhibitor Dorsomorphin dihydrochloride (Dd). Smad1/5/9 phosphorylation levels were unaffected by L745870 but were repressed by Dd (Figure 7E). These findings indicate that extracellular inhibition of DRD4 does not impair its ability to activate TGF‐β signaling, whereas blocking intracellular TGFBR1 prevents DRD4 from activating TGF‐β signals. Furthermore, the transwell assay revealed that Ga and Dd could suppress CRC migration and phosphorylation of Smad2 and Smad1/5/9 enhanced by DRD4 overexpression without affecting cell proliferation within a certain time frame (Figure S9G–I, Supporting Information). Meanwhile, Treatment with Ga and Dd significantly reduced the elevation of *SNAI1* and *JAG1* caused by DRD4 in DRD4‐overexpressing RKO cells (Figure S10A, Supporting Information) and DRD4 knockdown HCT116 cells (Figure S10B, Supporting Information). Finally, Ga and Dd were combined to pre‐treat DRD4 overexpression RKO cells, which were then injected into severe immunodeficient NCG mice via the tail vein (Figure 7F). The results indicated that RKO cells promoted CRC metastasis to livers and kidneys, and combination treatment with Ga and Dd markedly reduced the metastatic potential of DRD4‐overexpressing RKO cells to kidneys, but not to livers (Figure S10C–E, Supporting Information). IF assays confirmed the co‐expression of DRD4 and p‐smad2 in mouse liver and kidney metastases, which could be inhibited by Ga and Dd treatment (Figure S11A, Supporting Information). Moreover, the tail vein injection of DRD4‐overexpressing HCT8 cells also caused a reduction for the lung metastases following treatment with Ga and Dd (Figure 7G,H), while no reduction was observed in the kidneys (Figure S11B–D, Supporting Information). To further validate that TGFBR1 mediated DRD4‐induced metastasis, we knocked down TGFBR1 in DRD4‐overexpressing HCT8 cells (Figure 7I; Figure S12A, Supporting Information) and found that the migratory and invasive capabilities were suppressed in vitro (Figure S12B, Supporting Information). Meanwhile, TGFBR1 knockdown significantly reduced DRD4‐induced lung (Figure 7J,k) and kidney metastases in vivo (Figure S12C–E, Supporting Information). These in vitro and in vivo data suggest that DRD4 promotes CRC metastasis through interacting with TGF‐β family receptors independent of extracellular TGF‐β ligand signaling pathway.

## 3 Discussion

**Figure 8:**
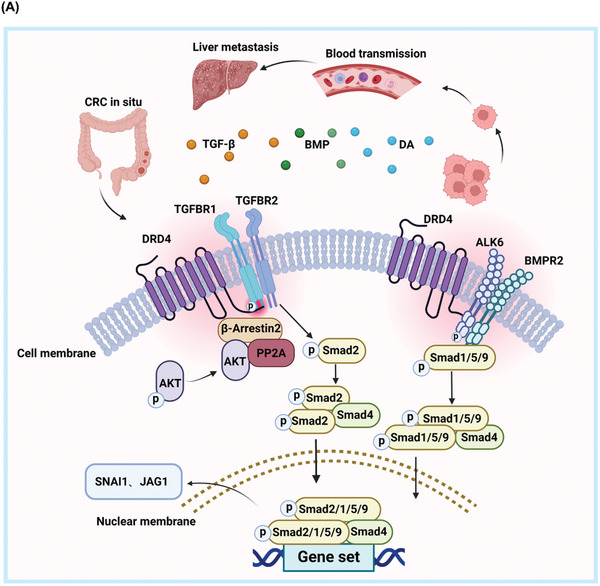
Schematic representation of constitutive DRD4 activates TGF‐β receptors independent of ligands to promote CRC metastasis. A) DRD4 activates the β‐Arrestin2/PP2A/AKT pathway independently of DA by interacting with TGF‐β receptors, thus triggering phosphorylation of Smad2/1/5/9 and promoting EMT through upregulation of *SNAI1* and *JAG1*, ultimately enhancing metastatic potential.

GPCR receptors play pivotal roles in tumor biology, influencing various biological functions such as cell proliferation, apoptosis resistance, angiogenesis, stemness, and metastasis.^[^ ^40^ ^]^ Consequently, targeting GPCR receptors has emerged as a promising strategy in cancer therapy. Our investigation not only find DRD4 is up‐regulated in CRC and negatively associated with prognosis, but also highlights the roles of DRD4 in CRC metastasis. DRD4 overexpression in CRC causes a constitutive activation of the β‐Arrestin2/PP2A/AKT pathway independent of DA, but this activation does not mediate the pro‐metastatic roles of DRD4. Meanwhile, DRD4 binds to multiple TGF‐β family receptors (including TGF‐β and BMP signaling receptors) on the cell membrane and increases Smad2 and Smad1/5/9 phosphorylation to form a complex with Smad4. Furthermore, nuclear translocation of Smad‐complex leads to the transcriptional upregulation of downstream target genes such as *SNAI1* and *JAG1*, further promoting EMT and metastasis (**Figure** **8**A).

As we know, DRD4 is conserved as a receptor for the neurotransmitter DA,^[^ ^37^, ^38^ ^]^ however, it remains unknow whether the pro‐metastatic role of DRD4 in CRC depends on classical DA pathway. Despite DA‐inducing intracellular calcium influx in CRC cells featuring heightened DRD4 expression, this phenomenon does not translate into observable changes in migration and invasion capabilities. Consequently, the phenotypic effects initiated by DRD4 cannot be solely attributed to the activation of the DA signaling pathway, despite DRD4 retaining its capability to bind to and activate this pathway. Thus, we hypothesize that the functionality of DRD4 in CRC may be intricately linked to cross‐talk with other signaling pathways. Previous studies confirmed that somatostatin (or somatotropin release‐inhibiting factor, SRIF), operating via specific G‐protein‐coupled membrane somatostain receptors (SSTRs), formed heterodimers with other GPCRs, such as the µ‐opioid receptor^[^ ^41^ ^]^ and DRD2,^[^ ^42^, ^43^ ^]^ thereby enhancing functional activity,^[^ ^44^ ^]^ even in the absence of ligands. The interplay between SSTR and DRD2 on the plasma membrane was implicated in promoting the proliferation of human prostate and lung cancer cell lines.^[^ ^45^ ^]^ This phenomenon, in which ligand‐independent receptor‐receptor interaction leads to functional changes, was also found in our findings.

The VNTR in the third intracellular loop region of DRD4 typically comprises four or seven tandem repeats, with fewer repeats potentially indicating functional deficiencies or disease predisposition.^[^ ^46^, ^47^ ^]^ Although DRD4 VNTR polymorphism has been associated with attention‐deficit hyperactivity disorder,^[^ ^47^ ^]^ abnormal personality traits,^[^ ^48^ ^]^ susceptibility to addiction,^[^ ^49^ ^]^ and Alzheimer's disease,^[^ ^50^ ^]^ no previous studies have linked the VNTR region of DRD4 to cancer. Our findings further elucidate that the pro‐metastatic functions of DRD4 in CRC are unrelated to the VNTR region. Additionally, studies have identified a palmitoylation site at the COOH‐terminus of DRD4, influencing receptor surface expression, endocytosis, signal transduction, and receptor function.^[^ ^51^, ^52^ ^]^ Protein S‐palmitoylation, a reversible post‐translational modification, plays a critical role in modulating the localization, stability, and function of various proteins within cells. This modification is crucial for the function of oncogenes (such as neuroblastoma RAS viral (v‐ras) oncogene and epidermal growth factor receptor) and tumor suppressors (such as scribble planar cell polarity protein and melanocortin 1 receptor).^[^ ^53^ ^]^ Our findings affirm that the COOH‐terminal domain of DRD4 is essential for its pro‐metastatic function in CRC, potentially through its association with the palmitoylation site.

DA can trigger immediate activation of the PI3K‐AKT signaling pathway, resulting in increased AKT phosphorylation. However, in CRC cells with elevated DRD4 expression, AKT phosphorylation remains persistently suppressed, which should be considered as a second‐stage activation of DRD4. Interestingly, we observed that DRD4 remained in a prolonged second‐stage activation state even in the absence of DA, termed as constitutive activation. Constitutive activation of dopamine receptors DRD1,^[^ ^54^ ^]^ DRD2,^[^ ^55^, ^56^ ^]^ DRD5^[^ ^52^, ^57^ ^]^ has been reported in relation to development and neurological disease, but the impact of the functional change on cancer progression and metastasis remains unclear. Therefore, the physiological implications of this “weak” and “continuous” activation state deserves further investigation.

We performed Gene Ontology pathway enrichment analysis on the RNA‐Seq results and identified a close association between the high expression of DRD4 and the TGF‐β signaling pathway. Notably, our findings indicate that DRD4 can modulate the activation of the TGF‐β signaling independently of its ligands. Co‐IP experiments confirmed that DRD4 could interact with 4 type I receptors and all 5 type II receptors of the TGF‐β family (formation of heterotetramers with combinations of 2 type I and 2 type II receptor molecules is essential for TGF‐β signaling pathway activation), indicating multiple possibilities for combinatorial activation and comprehensive TGF‐β family activation in CRC. Moreover, in the absence of ligands, DRD4 could interact with TGF‐β receptors to co‐activate, as demonstrated by PLA and IF experiments that showed intense binding between DRD4 and TGFBR1, and mutual binding even in the absence of TGFBR2. These results suggest a direct interaction between DRD4 and TGFBR1, leading to mutual activation. Otherwise, we found that the DRD4‐ΔC16 mutant does not alter the binding affinity with TGFBR1 and TGFBR2 but reduces the phosphorylation levels of both receptors. These results indicate that DRD4 modulates TGF‐β signaling, at least in part, by regulating the phosphorylation of TGFBR1 and TGFBR2. Thus, our findings suggest that DRD4 may regulate TGF‐β signaling through direct interaction and modulation of TGFBR1 and TGFBR2 phosphorylation. Meanwhile, in the absence of exogenous TGF‐β and BMP4, constitutively activated DRD4 interacted with TGF‐β receptors, resulting in increased levels of phosphorylated Smad2 and Smad159, as well as elevated expression of target genes *SNAI1* and *JAG1*. Given that the DA‐independent constitutive activation state of the DRD4 receptor still allowed it to bind to and activate ligands, we investigated TGF‐β receptors and found that the addition of recombinant TGF‐β and BMP4 still significantly activated the TGF‐β signaling pathway. These results demonstrate that both DRD4 and TGF‐β receptors maintain their respective abilities to bind to ligands and activate downstream signaling pathways in CRC.

Furthermore, our study reveals the ability of DRD4 to bind to TGFB1, but the precise mechanism of this interaction remains elusive. This interaction may be caused by faulty ligand recognition or indirect binding through TGF‐β receptors, but its occurrence underscores the significant correlation between DRD4 and the TGF‐β family. Besides, the activation of the TGF‐β signaling pathway induced by DRD4 exhibits deviations from the classical activation pathway. For example, we observed a significant inhibition of Smad3 phosphorylation by DRD4, which was different from classical TGF‐β signaling pathway activation. We demonstrated that Smad3 phosphorylation was inhibited by DRD4‐induced dephosphorylation of AKT. Conversely, recombinant TGF‐β stimulation increased Smad3 phosphorylation, highlighting disparities in the signaling pathways between DRD4‐induced and ligand‐induced activation.

## 4 Conclusion 

In conclusion, our findings elucidate that DRD4's interaction with TGFBR1/TGFBR2 constitutively activates the TGF‐β signaling pathway, thereby driving CRC metastasis. Consequently, DRD4 emerges as a potential biomarker for CRC progression and a promising pharmacological target for CRC therapy.

## 5 Experimental Section

### Clinical Materials and Ethics Statement

One hundred fourteen clinical CRC samples paraffin sections were collected from the Run Run Shaw Hospital, Zhejiang University, between 2018 and 2019. This study included 79 samples of CRC, 30 samples of adenomas, and 5 samples of metastases, and prognostic information was obtained from 69 cases. Informed consent was obtained from all the included patients. The study was approved by the Ethics Board of Biomedicine, Zhejiang University (2017027), China. The animal experiments were performed according to the Guide for the Care and Use of Laboratory Animals (The Ministry of Science and Technology of China, 2006). The use of animals in this study was approved by the Ethics Committee of Zhejiang University (reference number: ZJU20240236).

### Main Reagents and Cell Culture

Cell lines obtained from American Type Culture Collection include NCM460, RKO, HCT116, SW620, SW480, HT29, HCT8, DLD1, LoVo, and HEK293T were cultured with DMEM basic (1×) or RPMI Medium 1640 basic (1×). DMEM and 1640 were supplemented with 10% FBS and 1% penicillin‐streptomycin. Cells were cultured at 37 °C with 5% CO_2_ condition.

### Plasmid Construction and Transfection

This study employed pcDH, pcDNA3.1, and pLKO.1 plasmids for the construction of overexpression and knockdown protein vectors. The overall strategy involved amplification of the target fragments with sticky ends from the cDNA of 293T cells, using a high‐fidelity enzyme (Phanta Max Master Mix Dye Plus). These fragments were then subjected to homologous recombination with double‐enzyme‐cut plasmids to form intact plasmids. Transfection of CRC cell lines utilized the DNA transfection reagent Neofect (Genomtech, China), while transfection of 293T cells involved LipoD 293 reagent (SignaGen, USA), and siRNA transfection utilized GenMute siRNA Transfection Reagent (SignaGen, USA). Lentiviral packaging plasmids pMD2G and pXPAX2 were involved in lentivirus packaging, and the viruses were collected and filtered through 0.45 µm filters 48 h post‐transfection. Subsequently, depending on the requirement, different cell lines were infected, and puromycin (1 mg mL^−1^, 1–20 µL) or G418 (100 mg mL^−1^, 1–20 µL) was used for 4–7 days of selection to obtain the desired cell lines.

### Crispr‐Cas9 Technology

Two lentiviral sgRNA clones targeting the human DRD4 gene were procured from GeneCopoeia (Rockville, MD, USA). Lentiviruses were generated in HEK293 cells using the pCRISPR‐LvSG06, pVSVG, and pSPAX2 vectors. Subsequently, these lentiviruses were employed to transduce HCT116 and SW620 cells. Following ≈7 days of selection with puromycin, individual viable cells were isolated and cultured for ≈2 weeks. Finally, the knockdown efficiency of monoclonal cells was identified through DNA sequencing and immunoblotting. Primer sequences utilized are provided in Table S2 (Supporting Information).

### Cell Proliferation Assay

Cells were seeded in a 96‐well plate at a density of 2000–5000 cells 24 h prior. The following day, the cells were treated with drugs. At specific time points, 100 µL of fresh culture medium was replaced, and after incubating for 1–2 h following the addition of 10 µL of CCK8 reagent (Vazyme, China) to each well, the absorbance was measured at 450 nm detection wavelength to determine the OD values.

### Cell Migration and Invasion Assay In Vitro

Twenty‐four‐well transwell plates with 8‐µm pore size (Corning, USA) were employed for investigating cell migration and invasion. The filter membrane on the back of each chamber was uniformly coated with 20 µg mL^−1^ fibronectin (FN) from Sigma (Germany). After air‐drying, 50 µL of serum‐free culture medium, diluted 50 times, containing matrigel (Corning, USA) was added to the chambers, followed by incubation at 37 °C for 30 min. In the lower chambers of the 24‐well plate, 800 µL of complete culture medium with 10% serum was added. Cells (5×10^4^–2×10^5^) were diluted in 200 µL serum‐free culture medium and added to the chambers for 12–48 h of incubation. At specific time points, the medium in the chambers was aspirated, and the chambers were washed three times with PBS. Subsequently, they were fixed in 4% paraformaldehyde (LABLEAD, China) for 20 min. After three washes with PBS, the fixed chambers were stained in 0.001% crystal violet for 2–5 min, followed by rinsing with tap water. Excess cells inside the chambers were wiped off using a cotton swab. Finally, random fields were captured using an inverted microscope (Nikon ECLIPSE TS100, Japan), and ImageJ was utilized for cell quantification.

### Real‐Time Quantitative PCR (RT‐qPCR)

Cells were subjected to digestion using the Total RNA Isolation Reagent Trizol (Pufei, USA) for mRNA extraction. Subsequently, mRNA (1 µg) was reverse transcribed into cDNA using the reverse transcriptase HiScript II Q SuperMix for qPCR (+gDNA wiper, Vazyme, China). Specific cDNA synthesis and amplification, as well as polymerase chain reaction (PCR), were conducted following the respective product manuals. To quantify gene expression levels, β‐Actin was used as an internal reference, and the relative quantification of mRNA was calculated using the 2^‐ΔCt^ method. The primer pairs used are provided in Table S2 (Supporting Information).

### Western Blot

In brief, protein samples were separated on 8%, 10%, or 12% SDS‐page gels and subsequently wet‐transferred to nitrocellulose membranes (NC, Bio‐Rad, Hercules, USA). After blocking with 5% high‐protein milk for 1 h, the membranes were incubated with antibodies overnight at 4 °C and, on the following day, incubated with fluorescent secondary antibodies for 1 h in the dark. Exposures were performed using the Odyssey CLx Infrared Imaging System (LI‐COR, USA).

### Elisa Assay

Cell culture supernatants or cell lysates, collected and diluted in PBS gradients, were prepared. For cAMP ELISA assay, 1×10^6^ cells per 200 µL PBS with PMSF added was recommended. Standard wells, test sample wells, and blank wells were set up separately. For each well of the ELISA plate, 50 µL sample dilution was added, followed by the addition of working solution A. The plate was then incubated at 37 °C for 1 h and washed. Subsequently, working solution B was added, and the plate was incubated at 37 °C for another 1 h before washing. Chromogenic substrates were added to each well, and after terminating the reaction, absorbance was measured at a wavelength of 450 nm.

### Co‐Immunoprecipitation (Co‐IP)

FLAG beads (Sigma, Germany) were employed to capture proteins translated from exogenously transfected plasmids with a Flag tag in 293T cells. After elution, the eluted proteins were incubated with an HA‐tag primary antibody (CST, USA) to examine protein interactions. The Biolinkedin Classic IP/Co‐IP Kit (Biolinkedin, China) was used to capture endogenously expressed proteins in CRC RKO and HCT116 cells. Similarly, after elution, the eluted proteins were incubated with the corresponding binding protein antibodies to examine protein interactions.

### Immunofluorescent Staining

Cells were pre‐seeded in wells of glass‐bottom dishes, and the following day, they were fixed in 37 °C paraformaldehyde for 30 min. Permeabilization was achieved using 0.1% Triton‐X100 at 37 °C for 10 min, followed by blocking with 10% FBS for 30 min. The cells were then incubated with the primary antibody at 4 °C overnight. On the second day, after washing away the primary antibody, the cells were incubated with the secondary antibody (donkey anti‐mouse‐IgG‐Alexa Fluor 488, donkey anti‐rabbit‐IgG‐Alexa Fluor 546, or donkey anti‐goat‐IgG‐Alexa Fluor 647) from Invitrogen, USA, for 1 h. DAPI (Sigma, Germany) was used for nuclear staining and incubated for 20 min. Fluorescent signals were captured using a confocal microscope (FV2000, Olympus, Japan).

### Duolink PLA Fluorescence

Cells were pre‐seeded in wells of glass‐bottom dishes, and the following day, they were fixed in 37 °C paraformaldehyde for 30 min. Permeabilization was achieved using 0.1% Triton‐X100 at 37 °C for 10 min, followed by blocking with Duolink blocking solution at 37 °C for 60 min. After incubating with the primary antibody overnight at 4 °C and washing the next day, cells were incubated with PLA MINUS and PLUS probes for 1 h at 37 °C. Subsequent steps included washing, addition of the ligation enzyme with a 30‐min incubation at 37 °C, addition of the polymerase enzyme with a 100‐min incubation at 37 °C, and washing. The cells were then sealed with Duolink In Situ Mounting Medium containing DAPI and imaged using a laser confocal microscope (FV2000, Olympus, Japan).

### Calcium Probe

The day before, cells were seeded in immunofluorescence dishes. The following day, cells were incubated at 37 °C for 30 min with a solution containing 2 µm Fluo‐4 AM to load the fluorescence probe. After appropriate washing, an additional 30‐min incubation was carried out to ensure thorough esterase cleavage of Fluo‐4 AM within the cells, resulting in the formation of Fluo‐4, which then remained intracellular. Subsequently, after the addition of DA, fluorescence detection of Fluo‐4 was continuously performed for 15–30 min using the STORM Ultra High Resolution/Nikon A1 Confocal/Total Internal Reflection Microscope (N‐STORM/A1R, Nikon, Japan) to monitor changes in intracellular calcium ion concentrations.

### Immunohistochemical Analyses

The paraffin‐embedded tissue sections stored at −20 °C were brought to room temperature. Subsequently, they were placed in a 60 °C oven for 1 h to completely dissolve the paraffin on the slices. The paraffin sections were sequentially immersed in three fresh xylene solutions, each for 3 min. Following this, the sections were immersed in a gradient series of ethanol solutions with concentrations of 100%, 100%, 95%, and 75%, for 1 min each. They were then washed three times for 1 min each with PBS solution. The sections were immersed in a 3% H_2_O_2_‐methanol solution for 15 min, followed by a PBS wash. The paraffin sections were immersed in a citrate buffer, brought to a boil at 200 °C, and kept under constant pressure and temperature for 3 min. After cooling on ice to room temperature, the sections were washed with PBS solution. Tissues were outlined with a pencil, sealed with 10% fetal bovine serum, and incubated at room temperature for 30 min. After PBS wash, the primary antibody was applied and left overnight at 4 °C. On the next day, after washing with PBS, the secondary antibody (ultrasensitive enzyme) was applied for 1 h at room temperature. Following PBS wash, 100 µL of a diluted DAB solution was applied, and the reaction was timed for 3–5 min. The color change in the tissue was observed, and the slides were placed in tap water to stop the color development. The slides were rinsed under running water until the color became clear. Hematoxylin staining was performed for 30 s, and then the slides were rinsed under running water until the color became clear. Subsequently, the slides were sequentially immersed in a gradient series of ethanol solutions at concentrations of 75%, 95%, 100%, and 100%, each for 5 min. The sections were removed and air‐dried at room temperature on a slide rack. A drop of neutral resin was applied to the slides, avoiding bubble formation.

### Animal Metastasis Assays

All animal experiments were conducted in accordance with the protocol approved by the Zhejiang University Institutional Animal Care and Use Committee (Ethics Committee Number: 20742). Male NOD/ShiLtJGpt Prkdcem26Cd52Il2rgem26Cd22/Gpt mice aged 4 weeks, denoted as NCG (GemPharmatech, China), were intravenously injected with 1×10^6^ RKO or HCT8 cells. Metastatic tissues were isolated for histological analysis at 5–8 weeks. Spleen‐liver metastasis experiments were conducted using 5‐week‐old male nude mice. Under anesthesia, 1×10^6^ HCT116‐Mock and HCT116‐KD cells were orthotopically injected into the spleen. After 2 months, spleen and liver tissues were dissected for histological analysis.

### RNA‐Seq

Total RNA was extracted from HCT116‐mock, HCT116‐KD‐DRD4, SW620‐Mock, and SW620‐KD cells, and RNA sequencing and analysis were performed by RiboBio (Guangzhou, China). The construction of cDNA libraries was carried out using the Illumina TruSeq RNA Sample Preparation Kit (Illumina, San Diego, CA, USA). Individual RNA sequence libraries were pooled based on their respective sample‐specific 6 bp adapters, and sequencing was performed on the Illumina HiSeq 3000 sequencer with 150bp/paired‐end reads. Differential gene expression analysis was conducted using the Cuffdiff program within the Cufflinks software package. Genes with padj ≤0.05 and fold change 1 or fold change ≤‐1 were defined as differentially expressed genes, serving as candidate genes for further analysis and qPCR validation. The accession number for sequencing data was PRJNA1109554.

### Statistical Analyses

GraphPad PRISM and SPSS Statistics were used for statistical analysis. Normally distributed data were subjected to analysis using Student's *t*‐test, while non‐normally distributed data were analyzed using the non‐parametric Mann–Whitney U‐test. Comparison of two or more groups of data was analyzed using one‐way ANOVA or two‐way ANOVA. The data are presented as mean ± SD. Significance values are directly provided in the figures; “ns” indicates not significant.

## Conflict of Interest

The authors declare no conflict of interest.

## Author Contributions

Y.Z. performed the experiments, processed the data, and drafted the manuscript. J.T. collected clinical samples, performed IHC scoring, and gathered prognosis information for patients. M.W. analyzed the relationship between neurotransmitter receptors and prognosis. H.Z. provided experimental guidance and revised the manuscript. M.L. contributed to the experimental design.

## Ethical Statement

Informed consent was obtained from all the included patients. The study was approved by the Ethics Board of Biomedicine, Zhejiang University (2017027), China. The animal experiments were performed according to the Guide for the Care and Use of Laboratory Animals (The Ministry of Science and Technology of China, 2006). The use of animals in this study was approved by the Ethics Committee of Zhejiang University (reference number: ZJU20240236).

## Supporting information



Supporting Information

## Data Availability

The RNA‐seq data generated in this study (HCT116‐KD) have been deposited to the NCBI database under accession code PRJNA1109554.
