# Supplementary material for: DRD4 Interacts with TGF‐β Receptors to Drive Colorectal Cancer Metastasis Independently of Dopamine Signaling Pathway
Source: Adv Sci (Weinh). 2024 Dec 16;12(6):2413953. doi: 10.1002/advs.202413953 (PMC11809390; doi:10.1002/advs.202413953)
Supplement: Supplementary file 1 — Supporting Information [file ADVS-12-2413953-s001.docx]

**DRD4 Interacts with TGF-β Receptors to Drive Colorectal Cancer Metastasis Independently of Dopamine Signaling Pathway**

*Yuan Zhou, Jinlong Tang,* *Menghan Weng, Honghe Zhang* ^*^*, Maode Lai* ^*^

**Supporting Information**
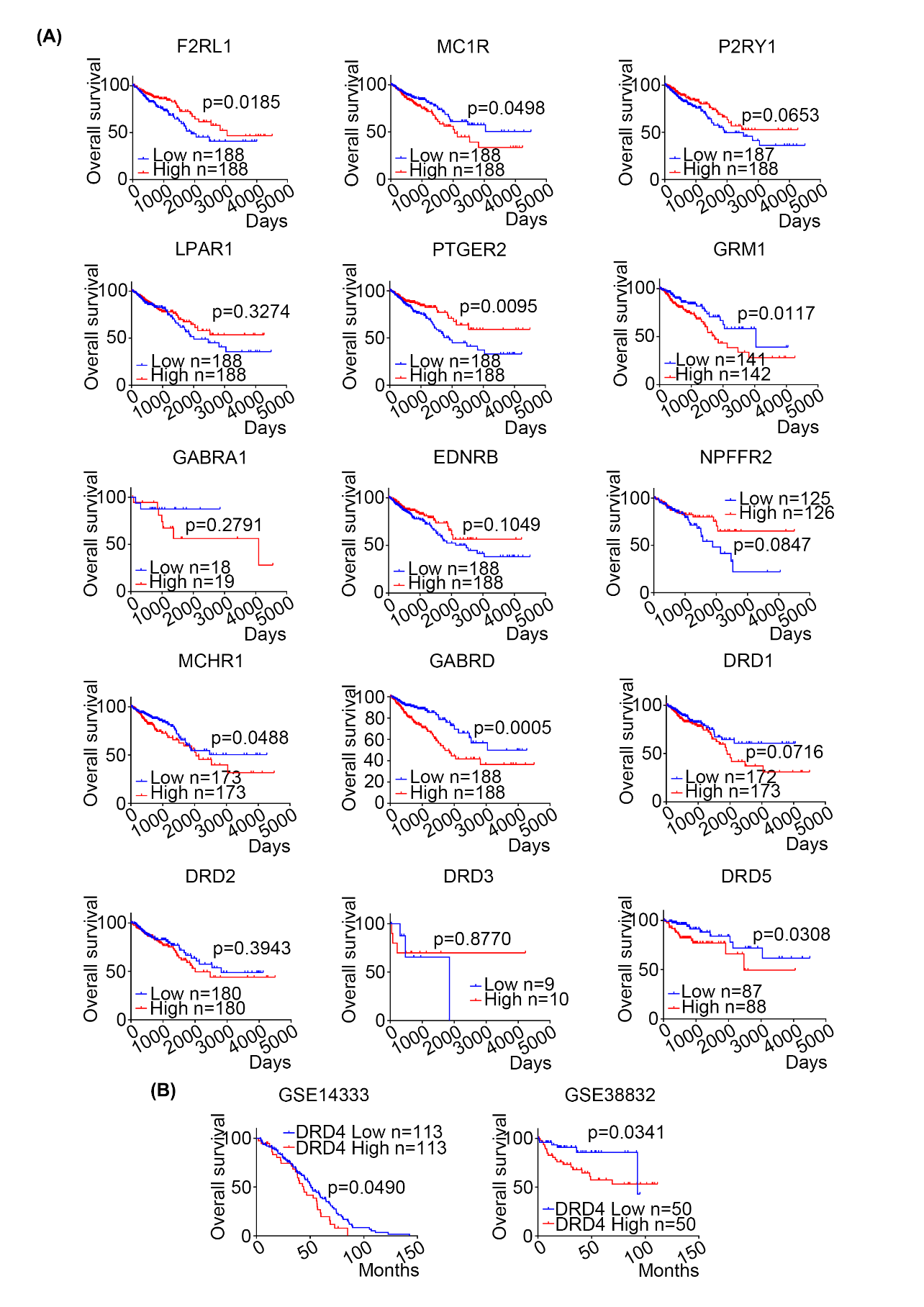


**Figure S1.** The relationship between neurotransmitter receptors and prognosis of CRC. (A). Kaplan-Meier plots of patients with CRC with high and low neurotransmitters (F2RL1, MC1R, P2RY1, LPAR1, PTGER2, GRM1, GABRA1, EDNRB, NPFFR2, MCHR1, GABRD, DRD1, DRD2, DRD3, and DRD5) expression. The numbers of samples are shown in the figure. (B). Kaplan-Meier plots of patients with CRC with high and low DRD4 expression in GSE14333 dataset and GSE38832 dataset. The numbers of samples are shown in the figure. Data are presented as mean ± SD; statistical significance was assessed by an unpaired t-test.


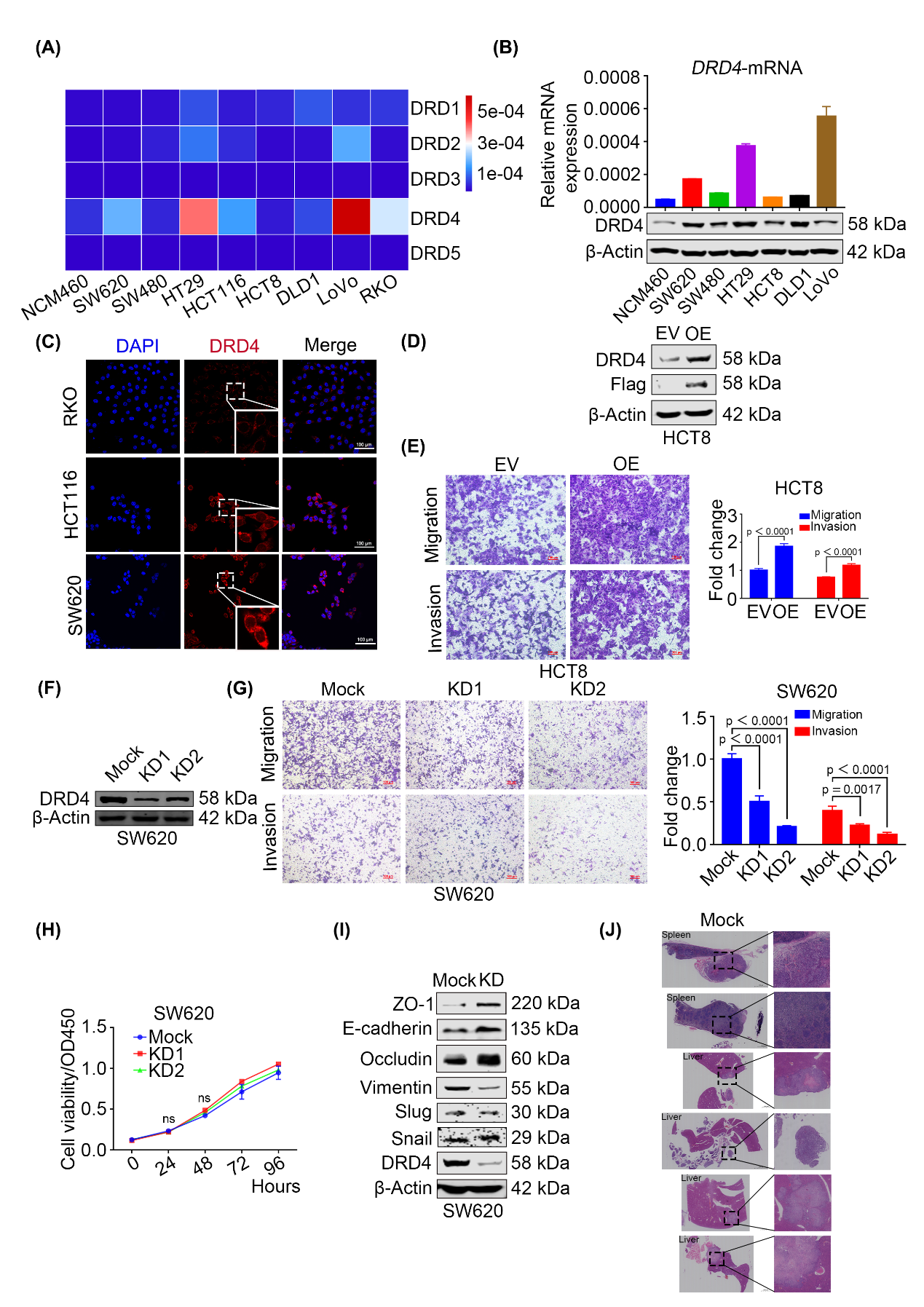


**Figure S2.** Expression and function of DRD4 in CRC. (A). The mRNA levels of *DRD1*, *DRD2*, *DRD3*, *DRD4* and *DRD5* in NCM460, SW620, SW480, HT29, HCT116, HCT8, DLD1, LoVo**,** and RKO cells were examined by RT-qPCR. β-Actin was run as an internal control. (B). The mRNA levels and protein expression of DRD4 in NCM460, SW620, SW480, HT29, HCT8, DLD1, and LoVo cells were examined by RT-qPCR and WB. β-Actin was run as an internal control. (C). Immunofluorescence staining of DRD4 in RKO, HCT116, and SW620 cells. Alexa Fluor 546-labeled DRD4 (red). Nuclei were stained with DAPI. Scale bars, 100 µm. (D). Protein levels of endogenic and exogenous Flag-tagged DRD4 were measured after stably transferred into pCDH-DRD4-N-3×Flag plasmid by WB and β-Actin was run as an internal control in HCT8 cells. (E). Transwell assay to investigate the migratory and invasive properties of DRD4 in overexpressed HCT8 cells. The histograms on the right show the quantification analysis results. (F). WB to detect the protein expression of DRD4 in DRD4-KD SW620 cells. β-Actin was run as an internal control. (G). Transwell assay to investigate the migratory and invasive properties of DRD4-KD SW620 cells. The histograms on the right show the quantification analysis results. (H). Effect of DRD4 knockdown on cell viability in SW620 cells within 96 h. CCK8 test wavelength was 450 nm. (I). Protein levels of EMT process markers were examined by WB in DRD4-KD SW620 cells. β-Actin was run as an internal control. (J). H&E staining of metastases in the spleens and livers of mice. Data are presented as the mean ± SD; statistical significance was assessed by two-way ANOVA.


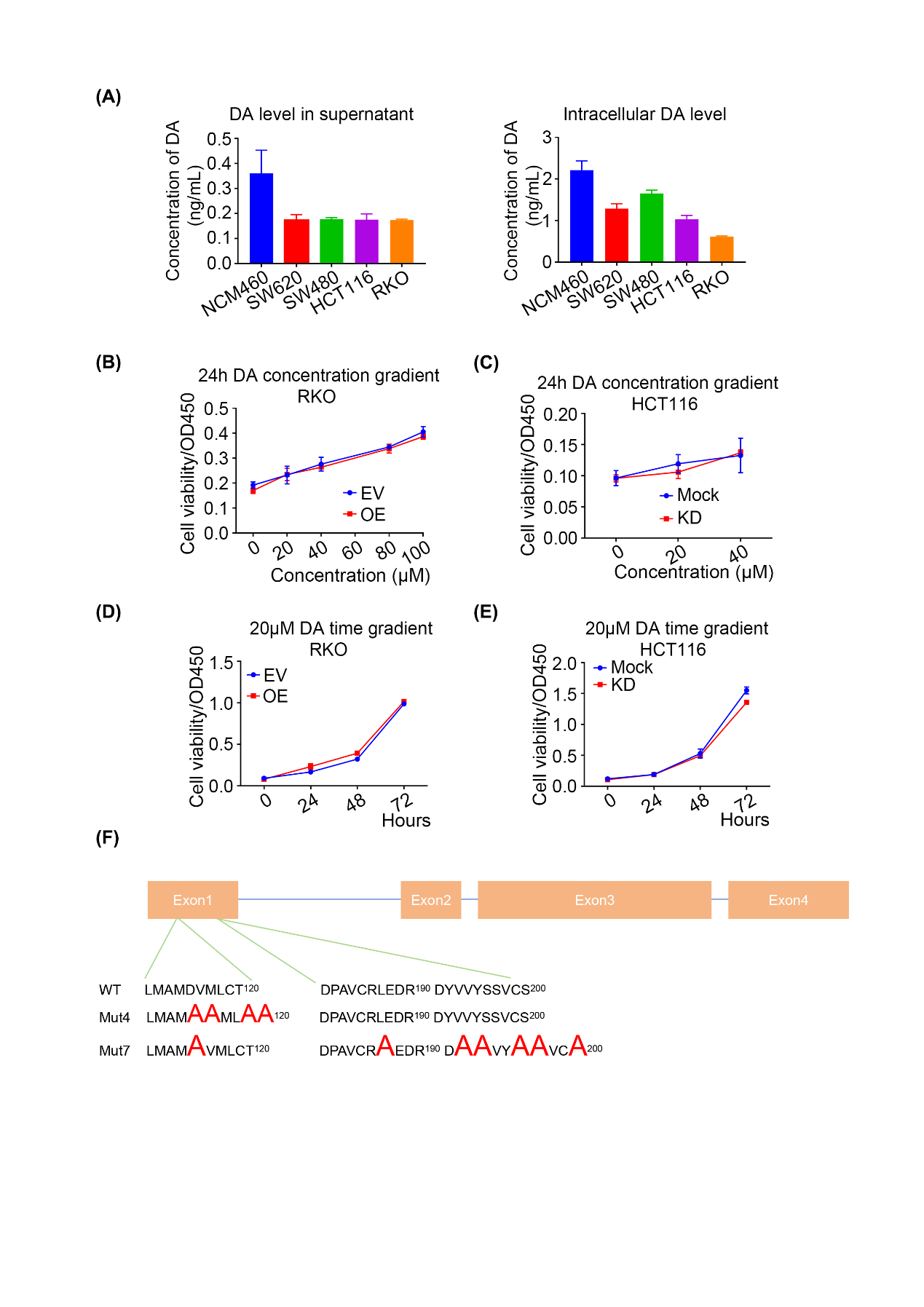
 **Figure S3.** The EMT-promoting function of DRD4 is independent of DA. (A). ELISA assay to detect the DA concentrations of supernatant and intracellular of NCM460, SW620, SW480, HCT116, and RKO cells. (B). Effect of DA concentration gradient on cell viability of DRD4-overexpressing RKO cells within 24 h. CCK8 test wavelength was 450 nm. (C). Effect of DA concentration gradient on cell viability of DRD4 knockdown HCT116 cells within 24 h. CCK8 test wavelength was 450 nm. (D). Effect of 20 μM DA on cell viability of DRD4-overexpressing RKO cells within 72 h. CCK8 test wavelength was 450 nm. (E). Effect of 20 μM DA on cell viability of DRD4 knockdown HCT116 cells within 72 h. CCK8 test wavelength was 450 nm. (F). Schematic diagram of DA binding sites mutation. Data are presented as the mean ± SD; statistical significance was assessed by one-way ANOVA or two-way ANOVA.


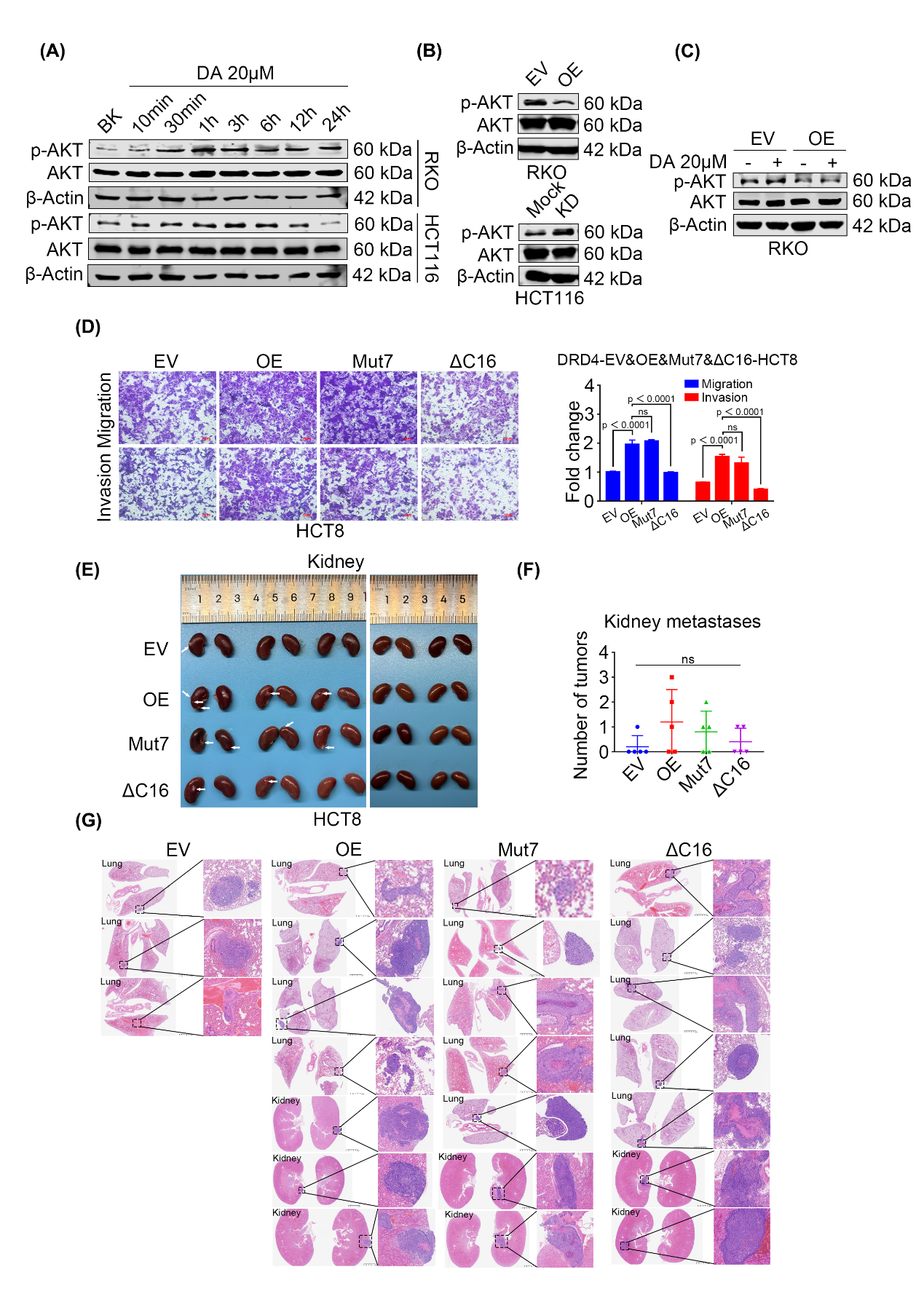
 **Figure S4.** The EMT-promoting function of DRD4 depends on the COOH terminal. (A). Protein levels of phosphorylated AKT (p-AKT) and total AKT were detected by WB in RKO and HCT116 cells within 24 h of treatment with 20 μM DA. Total AKT was run as an internal control. (B). WB to detect the p-AKT in DRD4-EV and DRD4-OE RKO cells, as well as DRD4-Mock and DRD4-KD HCT116 cells. Total AKT was run as an internal control. (C). WB to detect the effect of 20 μM DA on p-AKT in DRD4-EV and DRD4-OE RKO cells. Total AKT was run as an internal control. (D). Transwell assay to investigate the migratory and invasive properties of DRD4 with full-length, DRD4-Mut7 and DRD4-ΔC16 in HCT8 cells. The histograms on the right show the quantification analysis results. (E). Representative images of metastatic kidneys. EV (n=5); OE (n=5); Mut7 (n=5); ΔC16 (n=5). The white arrows represent the tumors. (F). Statistical diagram of the number of metastatic tumors in the kidneys of NCG mice injected via tail vein. EV (n=5); OE (n=5); Mut7 (n=5); ΔC16 (n=5). (G). H&E staining of metastases of lungs and kidneys. Data are presented as the mean ± SD; statistical significance was assessed by two-way ANOVA or an unpaired t-test.


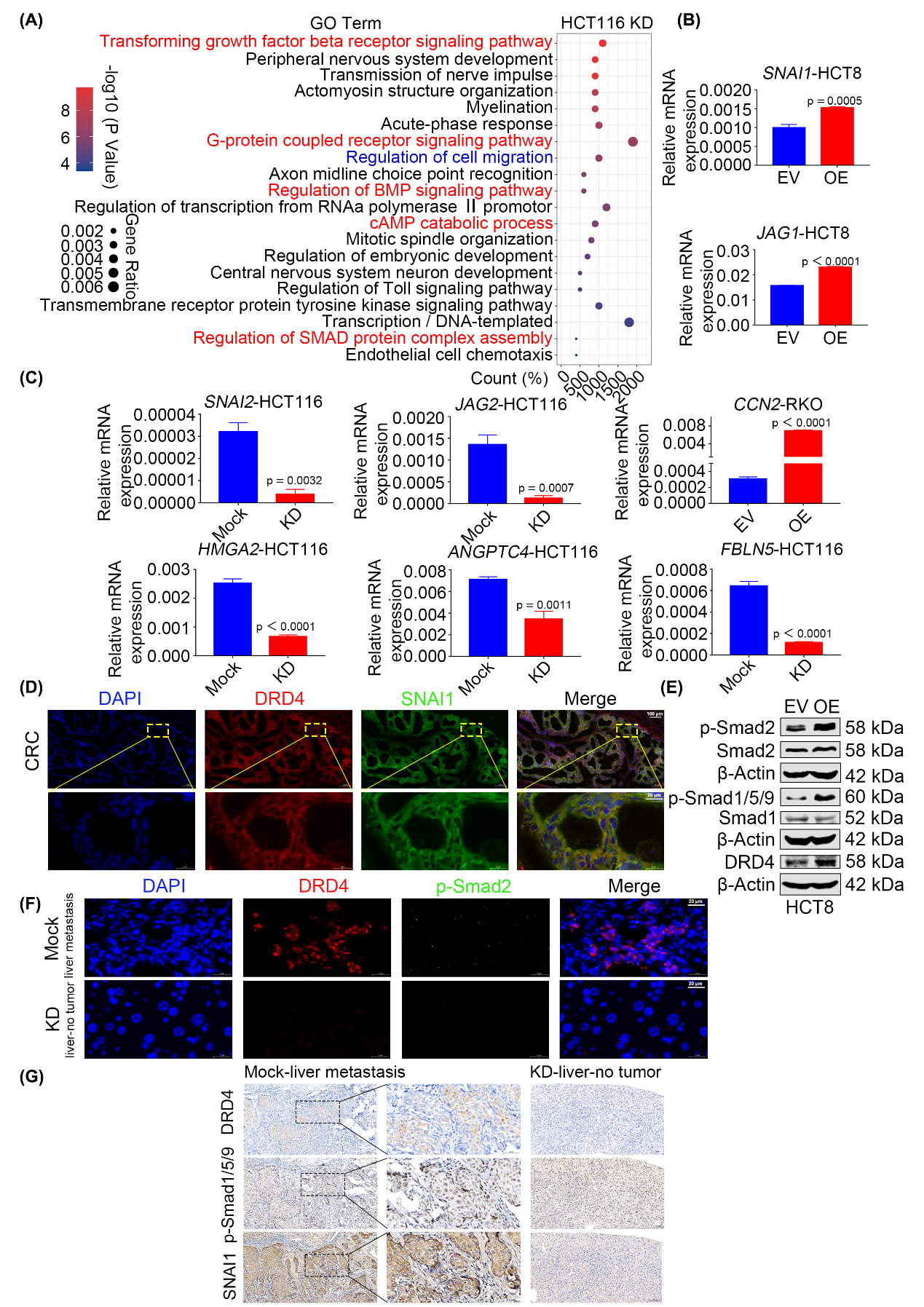


**Figure S5.** Constitutive activation of DRD4 is associated with the TGF-β signaling pathway. (A). Gene ontology (GO Biological Process) of DRD4-bound genes. (B). The mRNA levels of *SNAI1* and *JAG1* in DRD4-overexpressing HCT8 cells were examined by RT-qPCR. β-Actin was run as an internal control. (C). The mRNA expression of *SNAI2*, *JAG2*, *CCN2*, *HMGA2*, *ANGPTC4*, and *FBLN5* in DRD4 knockdown HCT116 cells or DRD4-overexpressing RKO cells were examined by RT-qPCR. β-Actin was run as an internal control. (D). Immunofluorescence staining for DRD4 (Alexa Fluor 546, red) and SNAI1 (Alexa Fluor 488, green). Nuclei were stained with DAPI. Scale bars, 100 µm; 20 µm. (E). WB to detect the levels of p-Smad2, Smad2, p-Smad1/5/9, Smad1, and DRD4 in the DRD4-overexpressing HCT8 cells. Smad2, Smad1, or β-Actin was run as an internal control. (F). Immunofluorescence staining for DRD4 (Alexa Fluor 546, red) and p-Smad2 (Alexa Fluor 488, green). Nuclei were stained with DAPI. Scale bars, 20 µm. (G). Representative images of standardized immunostaining for DRD4, p-Smad1/5/9, and SNAI1 in liver metastases of nude mice. Data are presented as the mean ± SD; statistical significance was assessed by an unpaired t-test.


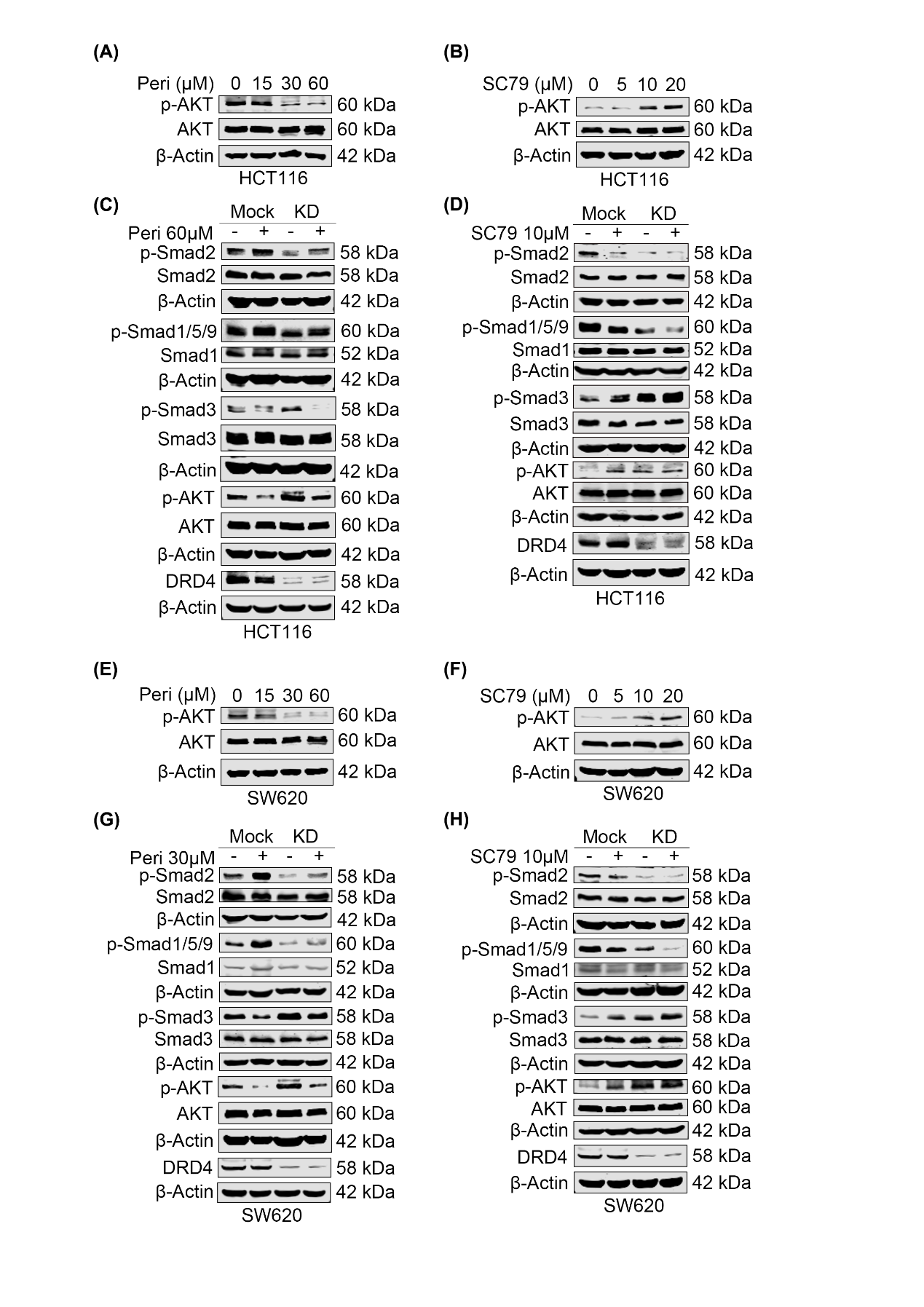
 **Figure S6.** The interaction between Smad3 and AKT leads to the decrease of p-smad3. (A). WB to detect the effect of concentration gradient of p-AKT inhibitor perifosine (Peri) on p-AKT in HCT116 cells at 24 h, respectively. AKT was run as an internal control. (B). WB to detect the effect of concentration gradient of p-AKT agonist SC79 on p-AKT in HCT116 cells at 24 h, respectively. AKT was run as an internal control. (C). WB to detect the effect of p-AKT inhibitor perifosine on p-Smad2, p-Smad1/5/9, p-Smad3, p-AKT, and DRD4 in the DRD4-KD HCT116 cells at 24 h, respectively. Smad2, Smad1, Smad3, AKT, or β-Actin was run as an internal control. (D). WB to detect the effect of p-AKT agonist SC79 on p-Smad2, p-Smad1/5/9, p-Smad3, p-AKT, and DRD4 in the DRD4-KD HCT116 cells at 24 h, respectively. Smad2, Smad1, Smad3, AKT, or β-Actin was run as an internal control. (E). WB to detect the effect of concentration gradient of p-AKT inhibitor perifosine on p-AKT in SW620 cells at 24 h, respectively. AKT was run as an internal control. (F). WB to detect the effect of concentration gradient of p-AKT agonist SC79 on p-AKT in SW620 cells at 24 h, respectively. AKT was run as an internal control. (G). WB to detect the effect of p-AKT inhibitor perifosine on p-Smad2, p-Smad1/5/9, p-Smad3, p-AKT, and DRD4 in the DRD4-KD SW620 cells at 24 h, respectively. Smad2, Smad1, Smad3, AKT, or β-Actin was run as an internal control. (H). WB to detect the effect of p-AKT agonist SC79 on p-Smad2, p-Smad1/5/9, p-Smad3, p-AKT, and DRD4 in the DRD4-KD SW620 cells at 24 h, respectively. Smad2, Smad1, Smad3, AKT, or β-Actin was run as an internal control.

#
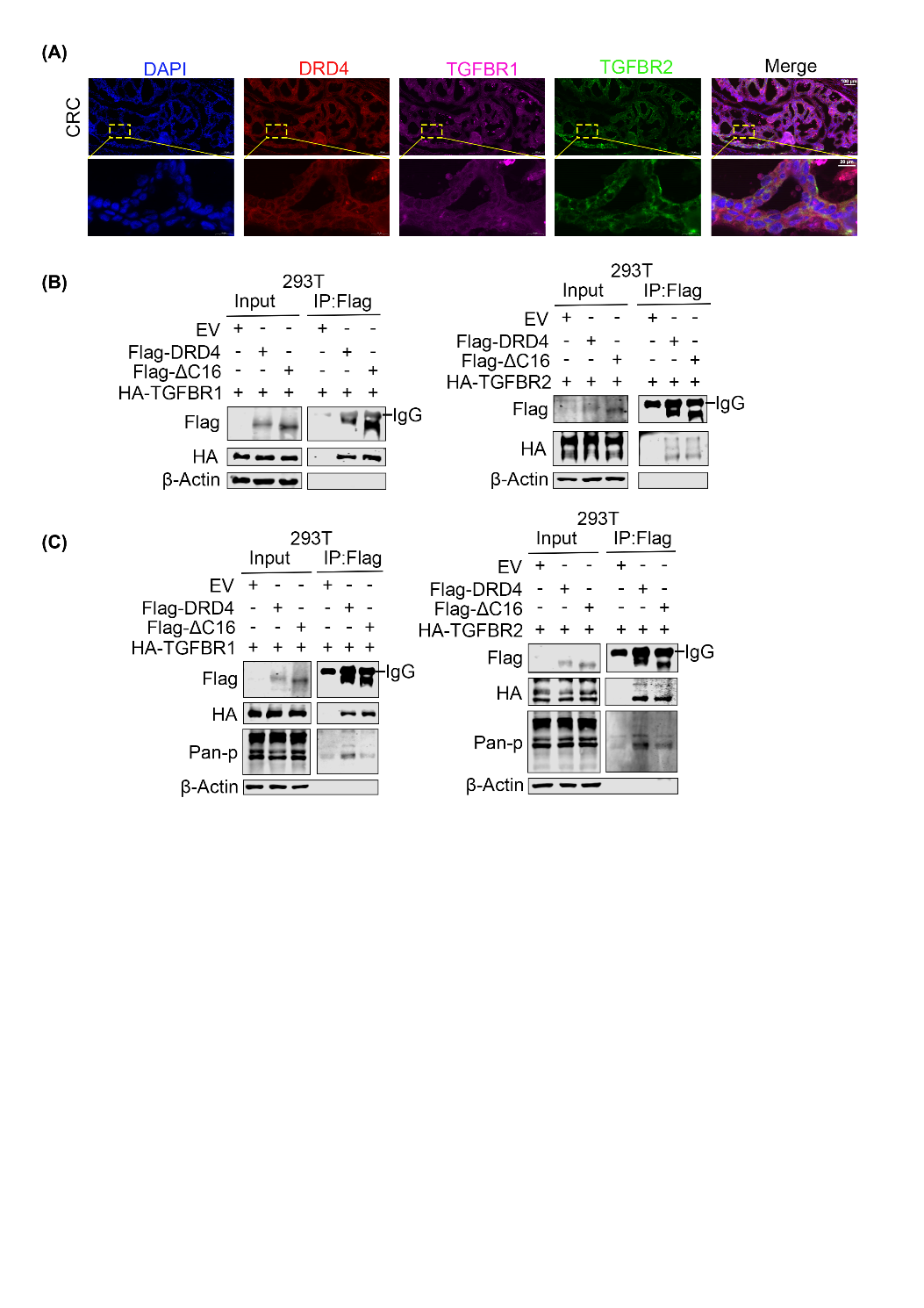
 Figure S7. DRD4 interacts with TGF-β receptors through phosphorylation (A). Immunofluorescence staining for DRD4 (Alexa Fluor 546, red), TGFBR1 (Alexa Fluor 647, purple) and TGFBR2 (Alexa Fluor 488, green) in clinical CRC sample. Nuclei were stained with DAPI. Scale bars, 100 µm; 20 µm. (B). Immunoblotting to detect the immunoprecipitation of exogenous Flag-tagged DRD4 and DRD4-ΔC16 with HA-tagged TGFBR1 or HA-tagged TGFBR2 by an anti-Flag antibody in 293T cells, respectively. (C). Immunoblotting to detect the immunoprecipitation of exogenous Flag-tagged DRD4 and DRD4-ΔC16 with HA-tagged TGFBR1 or HA-tagged TGFBR2 by an anti-Flag antibody in 293T cells, respectively. An anti-pan-phosphorylation antibody was used to detect the pan-phosphorylation.


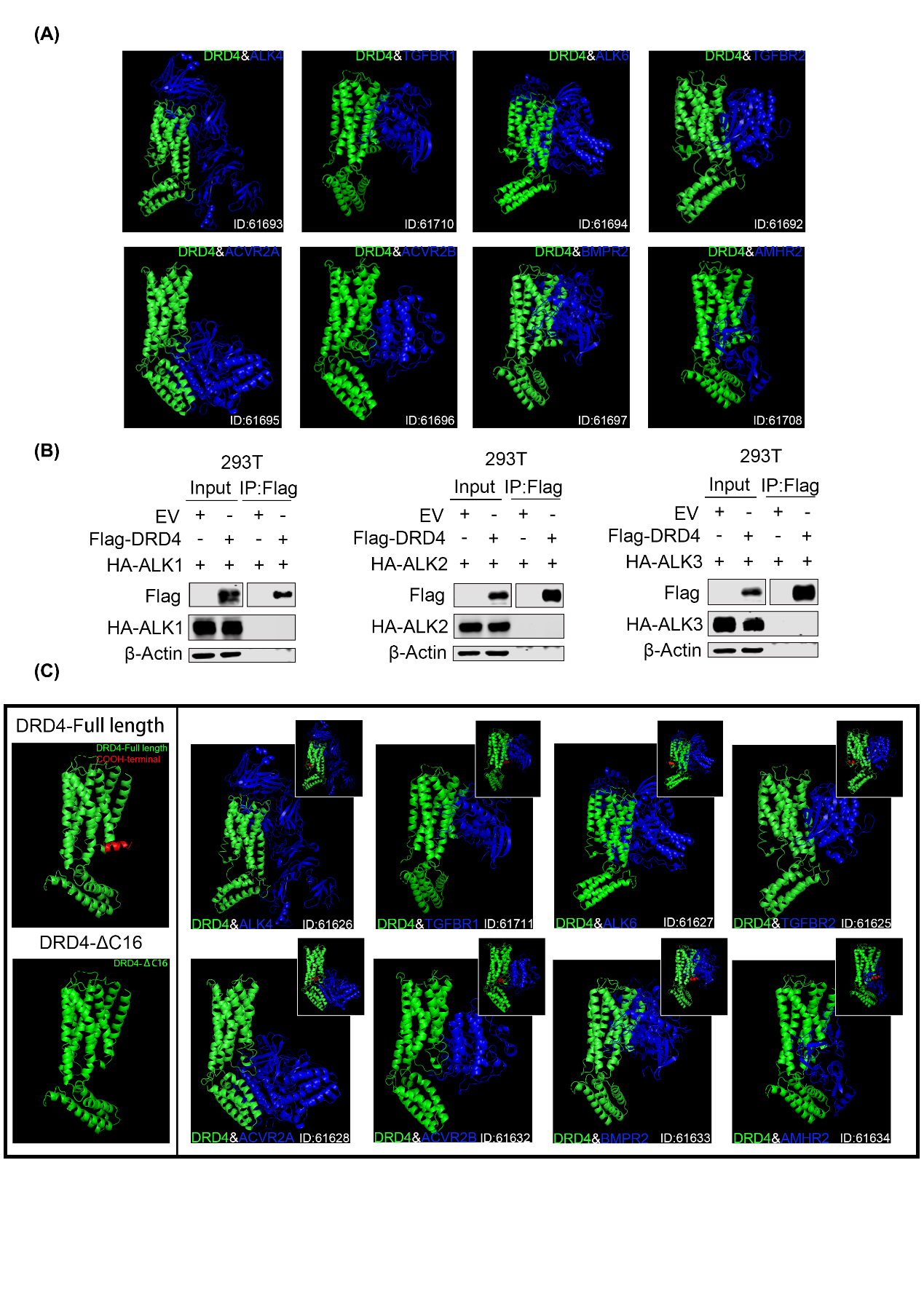


**Figure S8.** DRD4 interacts with TGF-β receptors. (A). Schematic diagram of predicting protein interactions between full-length DRD4 and 8 TGF-β family receptors. (B). Immunoblotting to detect the immunoprecipitation of exogenous Flag-tagged DRD4 with HA-tagged ALK1, ALK2, or ALK3 by an anti-Flag antibody in 293T cells, respectively. (C). Schematic diagram of predicting protein interactions between DRD4-ΔC16 and 8 TGF-β family receptors.


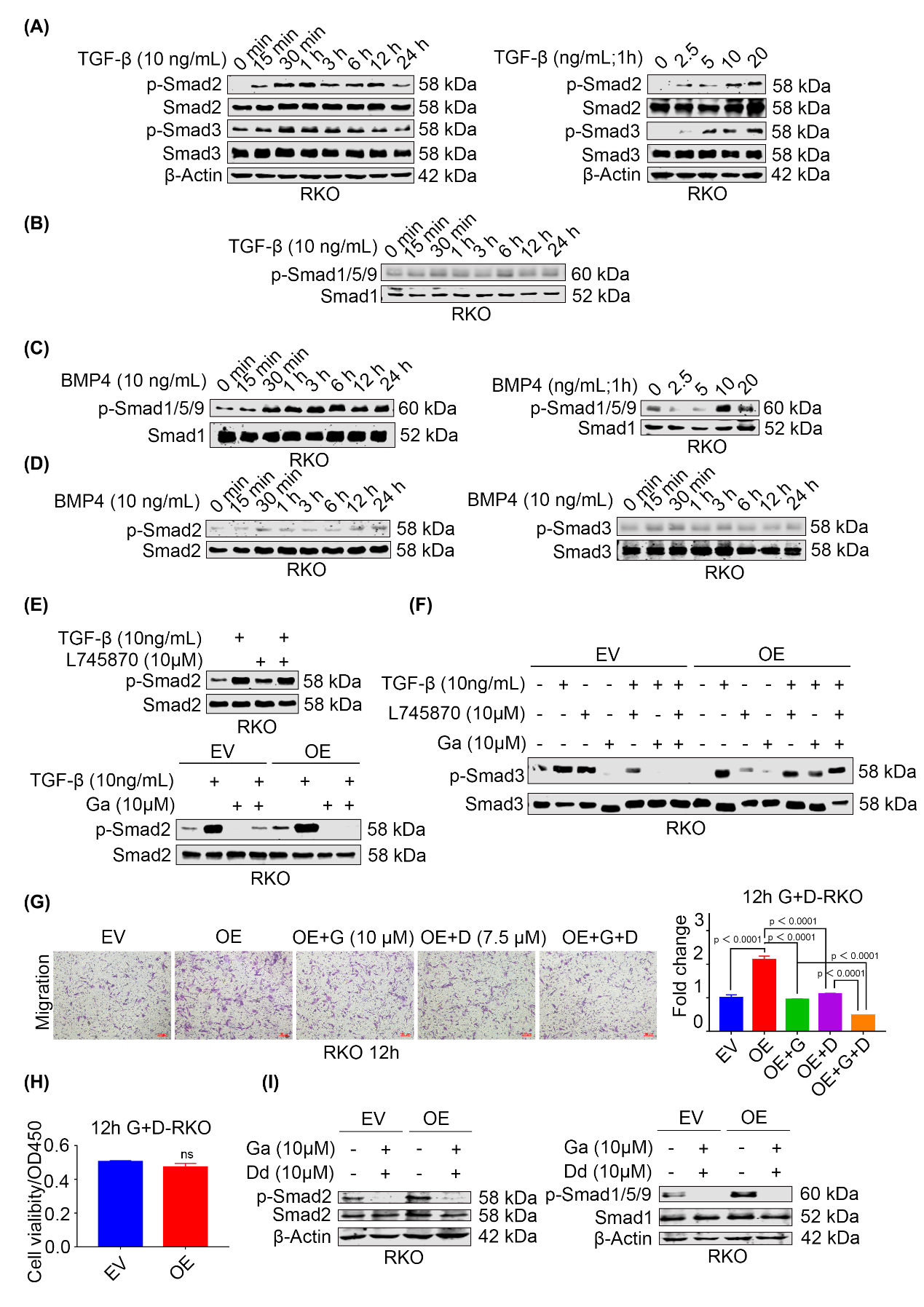


**Figure S9.** Inhibition of TGF-β receptors inhibits DRD4-induced metastasis. (A). WB to detect the effect of 10 ng/mL TGF-β on p-Smad2 and p-Smad3 in RKO cells with time and concentration gradients at 24 h, respectively. Smad2 or Smad3 was run as an internal control. (B). WB to detect the effect of 10 ng/mL TGF-β on p-Smad1/5/9 in RKO cells with time gradient at 24 h. Smad1 was run as an internal control. (C). WB to detect the effect of time and concentration gradients of BMP4 on p-Smad1/5/9 in RKO cells at 24 h. Smad1 was run as an internal control. (D). WB to detect the effect of 10 ng/mL BMP4 on p-Smad2 and p-Smad3 in RKO cells with time gradient at 24 h. Smad2 or Smad3 was run as an internal control. (E). WB to detect the effect of DRD4 inhibitor L745870 or TGF-β inhibitor Ga on p-Smad2 in DRD4-overexpressing RKO cells, respectively. Smad2 was run as an internal control. (F). WB to detect the effect of DRD4 inhibitor L745870 and TGF-β inhibitor Ga on p-Smad3 in DRD4-overexpressing RKO cells. Smad3 was run as an internal control. (G). Transwell assay to investigate the migratory properties of Ga and Dd in RKO cells at 12 h. The histograms on the right show the quantification analysis results. (H). The histograms showed the cell viability of Ga and Dd at 12 h. CCK8 test wavelength was 450 nm. (I). WB to detect the effect of Ga and Dd on p-Smad2 and p-Smad159 in DRD4-overexpressing RKO cells, which were used for tail vein injection. Smad2 or Smad1 was run as an internal control. Data are presented as the mean ± SD; statistical significance was assessed by one-way ANOVA or an unpaired t-test.


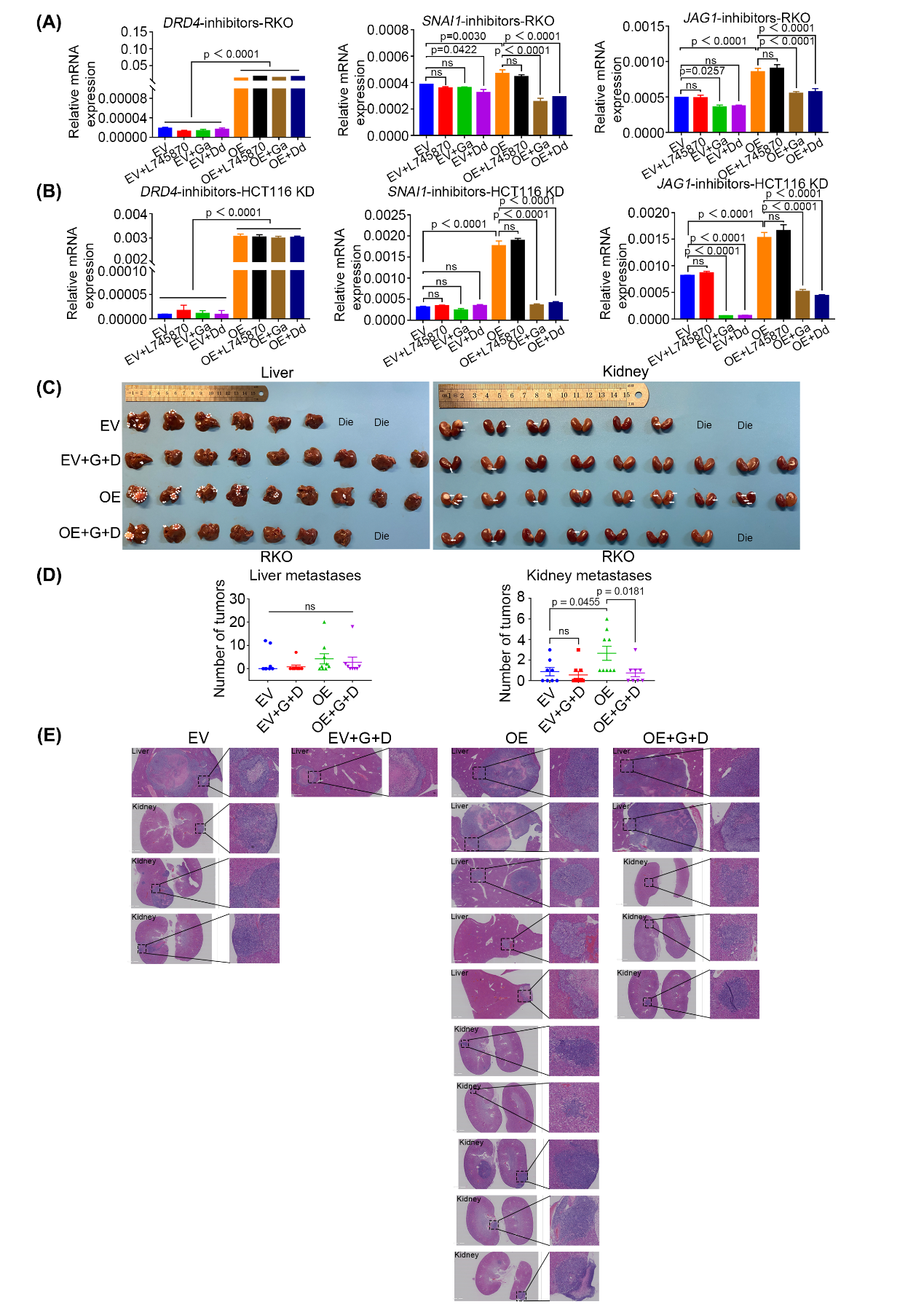


**Figure S10.** The effect of DRD4 and TGF-β inhibitors on CRC metastasis. (A). RT-qPCR to examine the effect of L745870, Ga, or Dd on mRNA levels of *DRD4*, *SNAI1*, and *JAG1* in DRD4-overexpressing RKO cells. β-Actin was run as an internal control. (B). RT-qPCR to examine the effect of L745870, Ga, or Dd on mRNA levels of *DRD4*, *SNAI1*, and *JAG1* when DRD4 was overexpressed in DRD4 knockdonw HCT116 cells. β-Actin was run as an internal control. (C). Representative images of metastatic livers and kidneys. EV (n=8); EV+G+D (n=9); OE (n=9); OE+G+D (n=8). The white circles and arrows represent the tumors. (D). Statistical diagram of the number of metastatic tumors in the livers and kidneys of NCG mice injected via tail vein. EV (n=8); EV+G+D (n=9); OE (n=9); OE+G+D (n=8). (E). H&E staining of metastases of livers and kidneys. Data are presented as the mean ± SD; statistical significance was assessed by two-way ANOVA or an unpaired t-test.


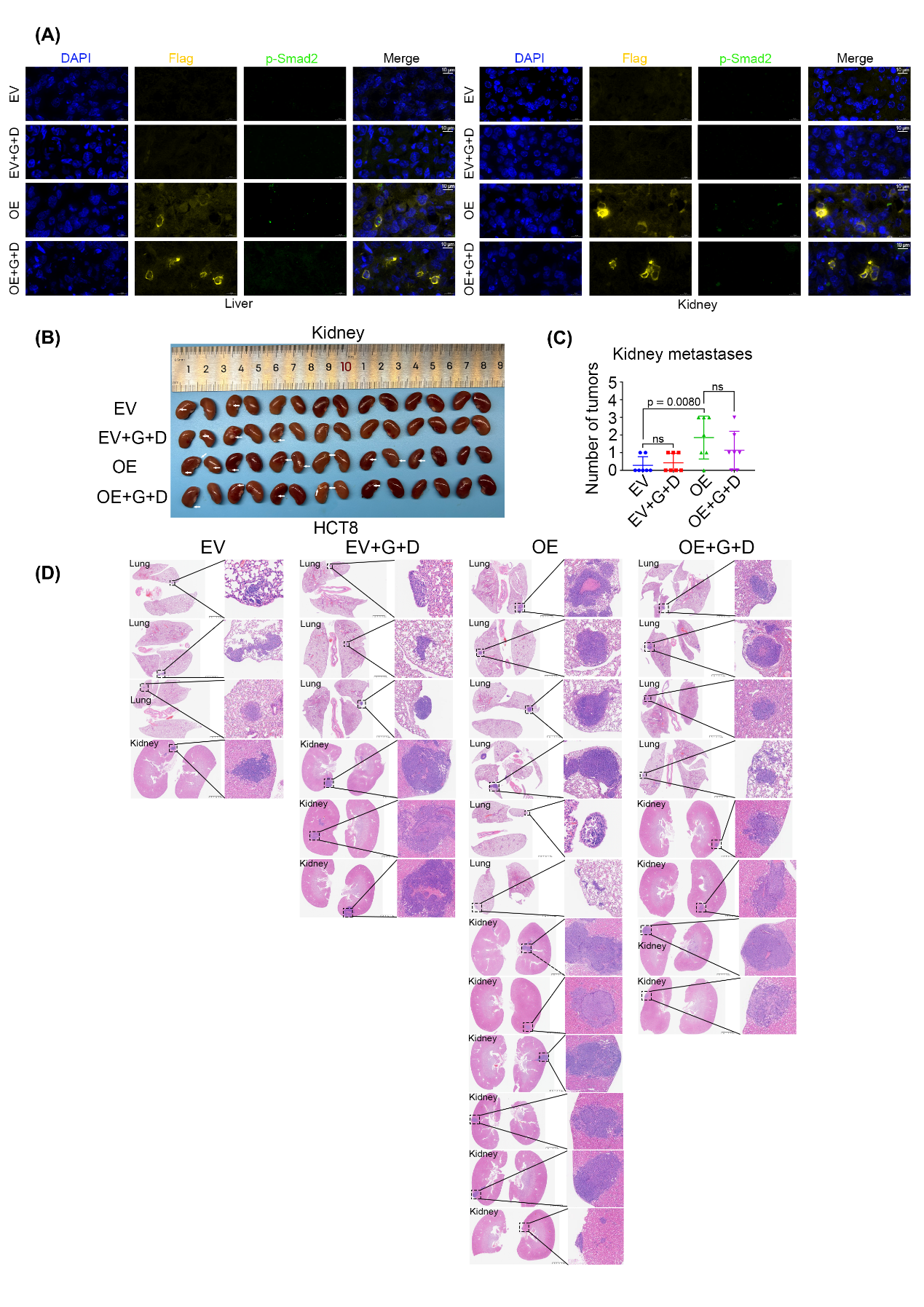


**Figure S11.** The effect of DRD4 and TGF-β inhibitors on CRC metastasis. (A). Immunofluorescence staining for exogenous Flag-tag (DRD4) (Alexa Fluor 647, yellow) and endogenic p-Smad2 (Alexa Fluor 488, green) in metastatic livers and kidneys. Nuclei were stained with DAPI. Scale bars, 10 µm. (B). Representative images of metastatic kidneys. EV (n=7); EV+G+D (n=7); OE (n=7); OE+G+D (n=7). The white arrows represent the tumors. (C). Statistical diagram of the number of metastatic tumors in the kidneys of NCG mice injected via tail vein. EV (n=7); EV+G+D (n=7); OE (n=7); OE+G+D (n=7). (D). H&E staining of metastases of lungs and kidneys. Data are presented as the mean ± SD; statistical significance was assessed by an unpaired t-test.


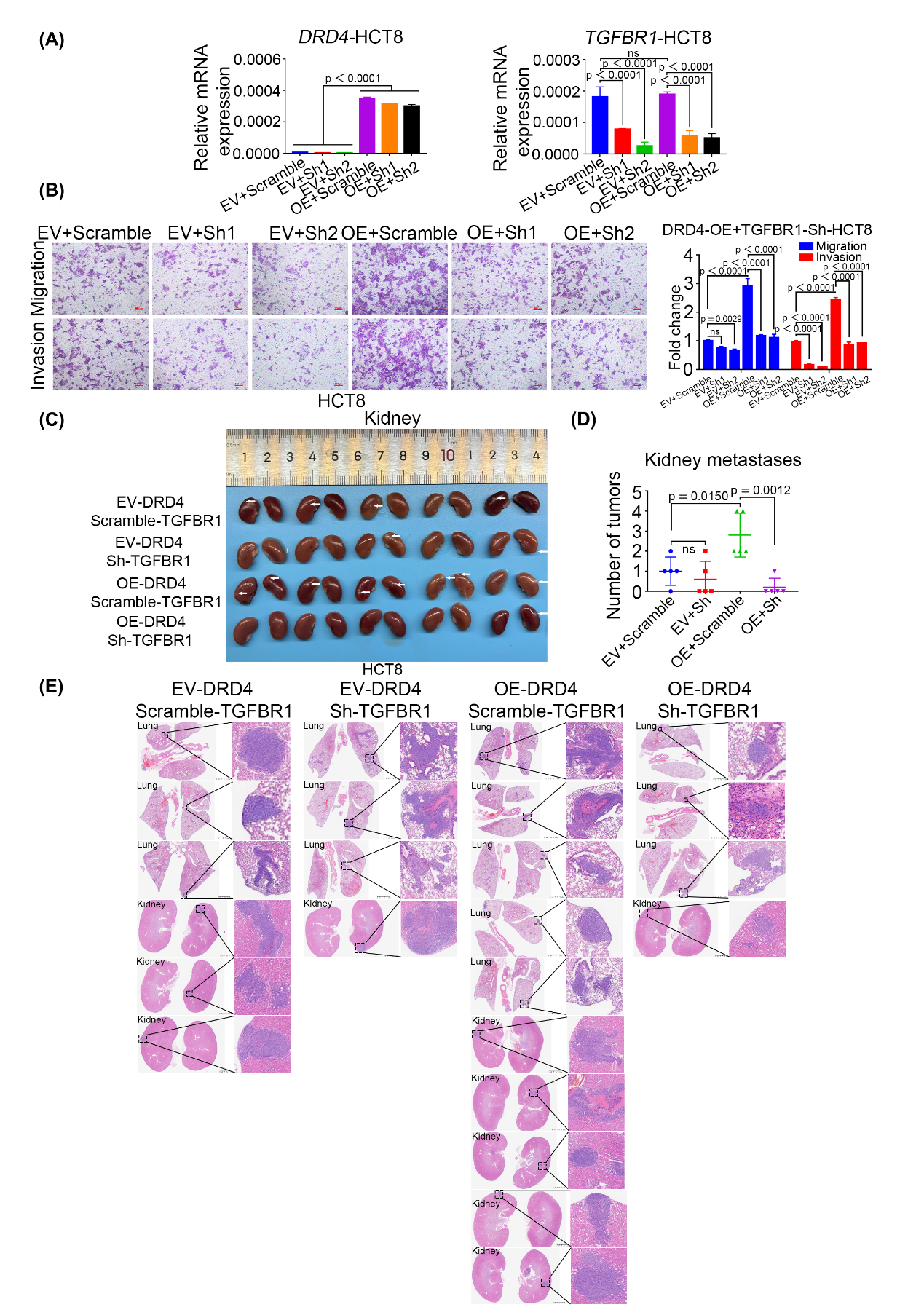


**Figure S12.** Effect of blocking the interaction of DRD4 and TGFBR1 on CRC metastasis. (A). RT-qPCR to examine the mRNA levels of *DRD4* and *TGFBR1* in DRD4-overexpressing and TGFBR1 knockdown HCT8 cells. β-Actin was run as an internal control. (B). Transwell assay to investigate the migratory and invasive properties of DRD4-overexpressing and TGFBR1 knockdown HCT8 cells. The histograms on the right show the quantification analysis results. (C). Representative images of metastatic kidneys. EV-DRD4+Scramble-TGFBR1 (n=5); EV-DRD4+Sh-TGFBR1 (n=5); OE-DRD4+Scramble-TGFBR1 (n=5); OE-DRD4+Sh-TGFBR1 (n=5). The white arrows represent the tumors. (D). Statistical diagram of the number of metastatic tumors in the kidneys of NCG mice injected via tail vein. EV+Scramble (n=5); EV+Sh (n=5); OE+Scramble (n=5); OE+Sh (n=5). (E). H&E staining of metastases of lungs and kidneys. Data are presented as the mean ± SD; statistical significance was assessed by two-way ANOVA or an unpaired t-test.

**Table S1.** Cox proportional hazards regression model and multivariate cox regression model to assess the correlation between the clinical features with the expression of DRD4 and overall survival.

| **Cox proportional hazards regression** | | | | **Multivariate cox regression** | |
| --- | --- | --- | --- | --- | --- |
| **Characteristic** | **Number** | ***P* value** | **HR^a)^ (95% CI****^b)^)** | ***P* value^c)^** | **HR^a)^ (95% CI^b)^)** |
| **Age [yr]** |  | 0.818 | 1.12 (0.43 ~ 2.93) |  |  |
| ≤60 | 27 |  |  |  |  |
| ＞60 | 42 |  |  |  |  |
| **Gender** |  | **0.047** | 0.23 (0.05 ~ 0.98) | 0.148 | 0.33 (0.08 ~ 1.48) |
| Male | 46 |  |  |  |  |
| Female | 23 |  |  |  |  |
| **Tumor location** |  | 0.068 | 0.32 (0.09 ~ 1.09) |  |  |
| Colon | 44 |  |  |  |  |
| Rectum | 25 |  |  |  |  |
| **Tumor size [cm]** |  | 0.365 | 0.51 (0.12 ~ 2.21) |  |  |
| ≤5 | 55 |  |  |  |  |
| ＞5 | 14 |  |  |  |  |
| **Histological classification** |  | 0.381 | 0.41 (0.05 ~ 3.06) |  |  |
| Canalicular adenoma | 60 |  |  |  |  |
| Others | 9 |  |  |  |  |
| **Differentiation** |  | 0.998 | 0.00 (0.00 ~ Inf) |  |  |
| Medium-low | 66 |  |  |  |  |
| High | 3 |  |  |  |  |
| **pTNM stage** |  | 0.139 | 2.02 (0.80 ~ 5.14) |  |  |
| Ⅰ/Ⅱ | 41 |  |  |  |  |
| Ⅲ/Ⅳ | 28 |  |  |  |  |
| **Lymphatic vascular invasion** |  | 0.072 | 2.40 (0.92 ~ 6.21) |  |  |
| Negtive | 49 |  |  |  |  |
| Positive | 20 |  |  |  |  |
| **Nervous invasion** |  | 0.284 | 1.67 (0.65 ~ 4.26) |  |  |
| Negtive | 44 |  |  |  |  |
| Positive | 25 |  |  |  |  |
| **Lymphnode metastasis** |  | 0.291 | 1.68 (0.64 ~ 4.43) |  |  |
| Negtive | 37 |  |  |  |  |
| Positive | 32 |  |  |  |  |
| **Distance metastasis** |  | **0.007** | 5.55 (1.60 ~ 19.28) | **0.036** | 3.90 (1.09 ~ 13.92) |
| Negtive | 52 |  |  |  |  |
| Positive | 17 |  |  |  |  |
| **DRD4 expression** |  | **0.002** | 1.28 (1.09 ~ 1.50) | **0.049** | 1.18 (1.01 ~ 1.40) |
| Negtive (Score = 0) | 28 |  |  |  |  |
| Positive (Score ≥ 1) | 41 |  |  |  |  |

^a)^ Hazard Ratio; ^b)^ Confidence Interval; ^c)^ Multivariate regression was carried out when *P* value of univariate analysis was ≤0.05.

Data available as of April 15, 2022.

Web link: https://shiny.medsta.cn/con5/

**Table S2.** Key resources

| **Reagents or Resource** | | **Source** | | **Catalog Number** |
| --- | --- | --- | --- | --- |
| Antibodies  Mouse monoclonal anti-SMAD1 | | Proteintech | | 66559-1-lg |
| Mouse monoclonal anti-SMAD2 | | Proteintech | | 67343-1-lg |
| Mouse monoclonal anti-SMAD3 | | Proteintech | | 66516-lg |
| Mouse monoclonal anti-TGFBR2 | | Proteintech | | 66636-1-lg |
| Mouse monoclonal anti-Phospho-AKT (Ser473) | | Proteintech | | 66444-1-lg |
| Mouse monoclonal anti-TGFβ RⅠ | | SANTA | | SC-518086 |
| Rabbit polyclonal anti-Beta Arrestin 2 | | Proteintech | | 10171-1-AP |
| Rabbit monoclonal anti-Phospho-Akt (Thr308) | | CST | | D25E6 |
| Rabbit polyclonal anti-AKT | | Proteintech | | 10176-2-AP |
| Rabbit polyclonal anti-PKA C-beta-specific | | Proteintech | | 55382-A-AP |
| Rabbit monoclonal anti-E-cadherin | | CST | | 3195 |
| ZO-1 (D6L1E) | | CST | | 13663 |
| Occludin (E6B4R) | | CST | | 91131 |
| Slug (C19G7) | | CST | | 9585 |
| Snail (C15D3) | | CST | | 3879 |
| DRD4 | | Guanggu | | NA |
| DRD4 | | OmnimAbs | | OM250419 |
| DRD4 | | CUSABIO | | CSB-PA967891 |
| DRD4 | | Immunoway | | YT1278 |
| Vimentin (D21H3) | | CST | | 5741 |
| Rabbit monoclonal anti-Phospho-Smad1 (Ser463/465)/Smad5 (Ser463/4650/Smad9 (Ser465/467) | | CST | | D5B10 |
| Phospho-SMAD1-S463/S465+SMAD5-S463/S465+SMAD9-S465/S467 Rabbit mAb | | Abclonal | | AP1518 |
| Rabbit monoclonal anti-Phospho-SMAD2 (Ser465/Ser467) | | CST | | E8F3R |
| Rabbit polyclonal anti-Smad2 (Ser467) | | Affinity | | AF3449 |
| Rabbit monoclonal anti-SMAD3 (phosphor S423+S425) | | Abcam | | AB52903 |
| Rabbit monoclonal anti-Smad3 | | CST | | C67H9 |
| Rabbit polyclonal anti-SMAD4 | | Proteintech | | 10231-1-AP |
| Rabbit monoclonal anti-TGF beta Receptor Ⅰ | | Abcam | | AB235578 |
| Rabbit polyclonal anti-TGFBR2 | | Proteintech | | 27212-1-AP |
| Recombinant Rabbit monoclonal anti-Jagged1 | | Huaan | | ET1702-63 |
| Jagged1 (D4Y1R) XP® Rabbit mAb | | CST | | 70109 |
| Rabbit polyclonal anti-SNAIL | | Huaan | | ER1706-22 |
| Snail Rabbit pAb | | Abclonal | | A5243 |
| Phospho-(Ser/Thr) Phe Antibody | | CST | | 9631S |
| Pan Phospho-Serine/Threonine | | Ab-mart | | T91067S |
| Monoclonal ANTI-FLAG® M2 antibody | | Sigma | | F1804-50UG |
| Rabbit monoclonal anti-HA-tag | | CST | | C29F4 |
| β-Actin | | Proteintech | | 66009-1-Ig |
| Alexa® Fluor 488 Donkey anti-Mouse IgG (H+L) | | ThermoFisher | | Cat#A-21202 |
| Alexa® Fluor 546 Goat anti-Rabbit IgG (H+L) | | ThermoFisher | | Cat#A-11010 |
| IRDye® 800CW Goat-anti-Rabbit Antibody | | LI-COR | | Cat#926-32211 |
| IRDye® 680CW Goat-anti-Mouse Antibody | | LI-COR | | Cat#926-68070 |
| IRDye® 800CW Donkey-anti-Goat Antibody | | LI-COR | | Cat#925-32214 |
| **Bacterial** | |  | |  |
| DH5a | | Vazyme | | C502-02 |
| **Biological Samples** | |  | |  |
| Patient samples | | Run Run Shaw Hospital Zhejiang University | |  |
| **Chemicals, Peptides, and Recombinant Proteins** | |  | |  |
| Puromycin | | Sigma | | Cat# P8833-10MG |
| Trizol | | Invitrogen | | Cat#15596018 |
| Lipofectamine2000 | | Invitrogen | | Cat#11668027 |
| LipoD293 | | SignaGen | | Cat # SL100668 |
| GenMute siRNA Transfection Reagent | | SignaGen | | Cat # SL100568 |
| Matrigel | | BD Biosciences | | Cat # 356234 |
| G 418 disulfate salt | | Sigm | | G 5013 |
| D-Luciferin, Potassium Salt | | Goldbio | | Cat#Luck-1G |
| **Critical Commercial Assays** | |  | |  |
| BCA protein assay | | ThermoFisher | | Cat#23225 |
| TALON® Metal Affinity Resin | | TAKARA | | Cat#635503 |
| ClonExpress-II One Step Cloning Kit | | Vazyme | | C112 |
| Mut Express II Fast Mutagenesis Kit V2 | | Vazyme | | C214 |
| Anti-FLAG® M2 Magnetic Beads | | Sigma | | Cat#M8823 |
| Duolink® In Situ Red starter | | Sigma | | DUO92105 |
| Duolink® In Situ Red | | Sigma | | DUO92101 |
| **Deposited Data** | |  | |  |
| Raw and analyzed data | | This paper | | NCBI BioProject ID |
| **Experimental Models: Cell Lines** | |  | |  |
| RKO | |  | | From ATCC |
| HCT116 | |  | | From ATCC |
| SW620 | |  | | From ATCC |
| HT29 | |  | | From ATCC |
| SW480 | |  | | From ATCC |
| HCT8 | |  | | From ATCC |
| DLD1 | |  | | From ATCC |
| LoVo | |  | | From ATCC |
| NCM460 | |  | | From ATCC |
| HEK293T | |  | | Chinese Academy of Sciences |
| **Experimental Models: Mice** | |  | |  |
| NCG | | Gempharmatech | | Gempharmatech.com |
| BALB/c-nude | | Gempharmatech | | Gempharmatech.com |
| **Oligonucleotides** | |  | |  |
| **Recombinant DNA** | |  | |  |
| pLKO.1 | | This paper | |  |
| pLKO.1-TGFBR1 | | This paper | |  |
| pMD2G | | This paper | |  |
| pSPAX2 | | This paper | |  |
| pcDNA3.1 (+) C-HA | | GenScript | |  |
| pcDNA3.1 (+) C-HA-DRD4 | | GenScript | |  |
| pcDNA3.1 (+) C-HA-ALK1 | | This paper | |  |
| pcDNA3.1 (+) C-HA-ALK2 | | This paper | |  |
| pcDNA3.1 (+) C-HA-ALK3 | | This paper | |  |
| pcDNA3.1 (+) C-HA-ALK4 | | This paper | |  |
| pcDNA3.1 (+) C-HA-ALK5 (TGFBR1) | | This paper | |  |
| pcDNA3.1 (+) C-HA-ALK6 | | This paper | |  |
| pcDNA3.1 (+) C-HA-ALK7 | | This paper | |  |
| pcDNA3.1 (+) C-HA-TGFBR2 | | This paper | |  |
| pcDNA3.1 (+) C-HA-ACVR2A- | | This paper | |  |
| pcDNA3.1 (+) C-HA-ACVR2B- | | This paper | |  |
| pcDNA3.1 (+) C-HA-AMHR2 | | This paper | |  |
| pcDNA3.1 (+) C-HA-BMPR2 | | This paper | |  |
| pcDNA3.1 (+) C-HA-Smad2 | | This paper | |  |
| pcDNA3.1 (+) C-HA-Smad3 | | This paper | |  |
| pcDNA3.1 (+) C-HA-TGFB1 | | This paper | |  |
| pcDH(+)N-Flag | | GenScript | |  |
| pcDH(+)N-Flag-DRD4 | | This paper | |  |
| pcDH(+)N-Flag-DRD4-ΔC16 | | This paper | |  |
| pcDH(+)N-Flag-DRD4-Mut7 | | This paper | |  |
| pcDH(+)N-Flag-DRD4-Mut4 | | This paper | |  |
| pcDH(+)N-Flag-ALK5 (TGFBR1) | | This paper | |  |
| pcDH(+)N-Flag-TGFBR2 | | This paper | |  |
| pcDH(+)N-Flag-AKT | | This paper | |  |
| pLentiCRISPR v2-DRD4 | | GenScript | |  |
| **Software and Algorithms** | |  | |  |
| GraphPad PRISM | | Open source | | https://www.graphpad.com |
| SPSS Statistics | | Open source | |  |
| GeneMapper ID-X | | Open source | | https://www.thermofisher.com |
| I STATISTICS | | Open source | | https://shiny.medsta.cn/con5/ |
| **Primer sequence** | Forward sequence (5’-3’) | | Reverse sequence (5’-3’) | |
| DRD4 | CCAACTCCTTCATCGTGAGC | | CCTGGACCTCGGAGTAGACG | |
| DRD1 | TGCCGTTATCAGGTTCCGAC | | CTGCCTTGGGGGTCATCTTT | |
| DRD2 | ACTATGCCACACTGCTCACC | | GCGCGTATTGTACAGCATGG | |
| DRD3 | CCCTGCAGACTACCACCAAC | | AACATCACAGCAAATGCGGC | |
| DRD5 | GGGGCAGTTCGCTCTATACC | | GATGACGCACAGGTTCAGGA | |
| JAG1 | GGCCGAGGTCCTATACGTTG | | TTAGGACTGCAGCCTTGTCG | |
| JAG2 | CCATGGGCTATTTCGAGCTG | | GGGTGGTATCGTTGTCCCAG | |
| SNAI1 | GCGAGCTGCAGGACTCTAAT | | TCCCAGATGAGCATTGGCAG | |
| SNAI2 | AGACCCCCATGCCATTGAAG | | GCAGTGCAGCTGCTTATGTT | |
| CCN2 | GTTTGGCCCAGACCCAACTA | | CATTGGTAACCCGGGTGGAG | |
| HMGA2 | CAGCAAGAACCAACCGGTGA | | AAGGCAACATTGACCTGAGCA | |
| ANGPTC4 | ACAGCAGGATCCAGCAACTC | | CTCCATGGTGCAGGCGG | |
| FBLN5 | TGCCAGGAATAAAAAGGATACTCAC | | ACTGGCGATCCAGGTCAAAG | |
| TGFBR1 | GTGACAGATGGGCTCTGCTT | | GCAATGGTAAACCAGTAGTTGGA | |
| Beta-ACTIN | CATGTACGTTGCTATCCAGGC | | CTCCTTAATGTCACGCACGAT | |
| TGFBR1-Sh1 | GATCATGATTACTGTCGATAA | | TTATCGACAGTAATCATGATC | |
| TGFBR1-Sh2 | GAAGTTGCTGTTAAGATATTC | | GAATATCTTAACAGCAACTTC | |
